# Developing consensus-based recommendations for the delivery of dementia services for the LGBTQIA+ community in the Republic of Ireland

**DOI:** 10.12688/hrbopenres.13505.1

**Published:** 2022-03-09

**Authors:** Megan H. Oglesby, Sinéad M. Hynes

**Affiliations:** 1 School of Health Sciences, National University of Ireland Galway, Galway, H91TK33, Ireland

**Keywords:** dementia, LGBTQIA+, older adults, healthcare access, healthcare recommendations, public and patient involvement

## Abstract

Background: The number of older LGBTQIA+ adults is set to rise significantly in the coming years. The rising numbers sit together with the rise in the number of people in Ireland diagnosed with dementia. In Ireland, no dementia-specific services exist for people from the LGBTQIA+ community. The aim of this research was to 1) identify the future needs that older LGTBQIA+ people and their care partners living in Ireland have in relation to dementia care service delivery; and to 2) develop consensus-based recommendations for dementia service provision in Ireland.

Methods: A six-phase consensus process was used to develop the lists of needs and recommendations: 1) development; 2) national survey; 3) interviews with key stakeholders; 4) international review of best practice; 5) consensus meeting; 6) final member checking. Participants, aged over 50, were based in Ireland, identified as a member of the LGBTQIA+ community or supported someone who is/was.

Results: Results are reported from the survey (n=49), individual interviews (n=8), and the consensus meeting (n=10). Participants have concerns related to identity management and suppression, creating an LGBTQIA+ affirmative ethos and workforce, and respect and safety. From the results and consensus process, a full list of ten prioritised needs and recommendations have been developed that focus specifically on dementia care in Ireland for the LGBTQIA+ community.

Conclusion: The older LGBTQIA+ community has identified essential priorities for improving healthcare access and safety. These priorities now need to be urgently implemented into clinical and dementia care services.

## Background

In 2011, Higgins and colleagues published the “Visible Lives” report that stated that “ *whilst some of the issues facing older LGBT people may be similar to those for all older people, there is a growing awareness of the need to identify the specific issues older LGBT people face.*” ( Higgins *et al*., 2011, Key Findings, p3). Many older people from the lesbian, gay, bisexual, transgender, queer/questioning, intersex, asexual + (LGBTQIA+) community have experienced discrimination and marginalisation in their lives. As homosexuality was not decriminalised in Ireland until 24 June 1993, ( Criminal Law [Sexual Offences] Act, 1993) many of the older LGBTQIA+ people living in Ireland came of age at a time when same-sex behaviour or gender non-conformity was severely stigmatised and criminalised. Countless people left Ireland or concealed their gender and/or sexual identity because they felt uncomfortable or unsafe. Many older LGBTQIA+ people feel increasingly vulnerable as they age and have significant worries related to preparation for ageing, in particular trans and gender non-conforming older adults ( Sharek *et al.*, 2015). This is often compounded by previous life experiences.

The number of older LGBTQIA+ adults is set to rise significantly in the coming years, with the number of older people in general rising ( Sheehan & O’Sullivan, 2020) at the same time as more people are revealing their gender identity or sexual orientation later in life. With that, the numbers of people living with dementia in Ireland is also on the increase ( Alzheimer Europe, 2020). There is estimated to be between 39,272 and 55,266 people with dementia in Ireland, which is an increase of 7752 new cases per year ( Pierse *et al.*, 2019). There is also some debate as to whether older people from sexual minorities are at an elevated risk of cognitive impairment ( Perales-Puchalt *et al.*, 2019), with recent robust research suggesting that the rates of cognitive impairment appear to be significantly higher among sexual minority older adults than among heterosexual older adults, even when sociodemographic factors are adjusted for ( Hsieh *et al.*, 2021).

Research has clearly shown that older LGTBQIA+ adults are less likely to engage with health services and community groups and are more likely to report poor general and mental health ( Fredriksen-Goldsen *et al*., 2015; Hoy-Ellis & Fredriksen-Goldsen, 2017; Wallace *et al.*, 2011). Some people articulate strong social support networks ( King & Cronin, 2016) but this is not the case for many ( Kim *et al.*, 2017), with increased levels of loneliness and isolation seen in this population ( Kuyper & Fokkema, 2010). It is also concerning that older LGBTQIA+ people do not engage with health services until a crisis situation, and 40% do not disclose their sexuality to their care providers specifically due to the possibility of discrimination ( Higgins *et al.*, 2011). Health inequalities can be seen in this community and yet in Ireland very little, if anything, has been done to address the lack of diversity in health care delivery for older adults ( Roe *et al.*, 2020). Internationally examples of good practice in relation to dementia and older adult services for LGBTQIA+ community exist – for example the UK Government published National LGBT Action Plan in 2018 and appointed a National Advisor for LGBT Health which appear to be having a positive impact on health and well-being of the older LGBTQIA+ community ( Opening Doors London, 2021). Nationally, there is an imperative need to ensure our health and care services are addressing the needs of under-served populations- for vulnerable groups such as older people from the LGBTQIA+ population. Building on recent recommendations in this area ( Roe *et al.*, 2020), this research aimed to:

1. Identify the future needs that older LGTBQIA+ people and their care partners living in Ireland have in relation to dementia care service delivery.
2. Develop consensus-based recommendations for dementia service provision in Ireland.

## Methods

Ethical approval for the research was granted by the National University of Ireland Galway Research Ethics Committee- Reference number 2021.05.010. Data was collected between July 2021 and December 2021. The Standards for Reporting Qualitative Research (SRQR; O’Brien *et al*., 2014) were followed in reporting the results and the SRQR checklist can be found in Extended Data.

### Design

The traditional Delphi consensus process (as described in Hsu & Sandford, 2007), which involves multiple iterations with highly trained and specialised Delphi participants, is not well suited for a population of people with dementia. The consensus process used here was adapted to ensure accessibility to people with dementia, older people and care partners ( Morbey *et al.*, 2019). The research included older LGTBQIA+ people with and without dementia through the research process (with guidance from Swarbrick *et al.*, 2019). There was substantial member involvement throughout (see section on public and patient involvement; PPI). A six-phase process took place in order to reach consensus on recommendations and prioritised needs. 

### Procedure

Six phase consensus process:

*Phase 1 – Development*. The PPI advisory group for the research was created as a first step. A questionnaire was then adapted, from Fredriksen-Goldsen & Kim (2017), following a thorough review of literature. PPI members decided on the inclusion of items, length of questionnaire, format of questionnaire, rating scales and accessibility issues (to include language used). The research team and PPI group worked in close collaboration to ensure final questionnaire distributed was appropriate for both people with dementia and LGBTQIA+ community.

*Phase 2 - National survey of older LGBTQIA+ people and care partners*. The questionnaire, adapted and piloted in Phase 1, was distributed. Postal and online completion options were used, Participants also had the option of completing the questionnaire over phone/video call.

*Phase 3 - Interviews with key stakeholders* - Older LGTBQIA+ adults and care partners were interviewed using an online platform (but provided with the option of a telephone interview if preferred). Those who indicated an interest in doing so, through the online questionnaire, were invited to an individual interview. The aim of the interviews was to gain more in-depth information on needs that were not captured in the survey and to further discuss future care needs.

*Phase 4 - International review of best practice*. This involved a review of literature in the area, as well as policies and frameworks developed in other countries. As part of this phase, we also interviewed international experts working in dementia care for LGBTQIA+ community. The aim was to develop a picture of what best practice in the area looked like internationally.

*Phase 5 - (Virtual) consensus meeting.* The aim of the consensus meeting was to agree a set of needs and recommendations. The consensus meeting involved the PPI advisory group, those who took part in the individual interviews, and representatives from voluntary and healthcare backgrounds working with people with dementia. A purposeful sampling strategy was used to ensure a diverse group with varied experiences and backgrounds. Because of the virtual nature of the meeting and because participants may have been experiencing cognitive impairment, the number of participants included in the meeting was kept low (maximum 10).

The meeting used a modified nominal group technique to ensure participation of all members and was guided by similar research in the area ( Brett *et al.*, 2017; Keegan *et al.*, 2021; Schneider *et al.*, 2016). The nominal group technique was used because it reduces the burden on participants and results can be obtained quickly and presented back to the group ( McMillan *et al.*, 2016). This was deemed appropriate considering the additional scoping work (phases 2–4) that preceded the consensus meeting.

Results of phases 2, 3 and 4 were presented to the group, along with the needs and recommendations that came from the research. Sli.do, an online polling tool, was used to facilitate the adding, voting, and ranking of items. Because Sli.do automatically calculates the ranking, it was possible to work through all the stages of the meeting in real time (see stages below):

As recommended and used elsewhere (e.g. Keegan *et al.*, 2021), the modified nominal group technique used consisted of four stages- 1) silent generation when participants had the opportunity to think about any additional items they wanted to add; 2) round robin where participants added those items they felt were missing- this was done anonymously; 3) clarification – participants were provided with the opportunity if they chose to discuss any new items or seek clarification; 4) private voting and ranking of items. There were two rounds of voting on both the “Needs” and “Recommendations” once the additional items were added. The first round of voting was to rank the importance of each item. From this the “Top 10” list of items were identified and participants then had the chance to rank the items in order of importance (in Phase 6).

*Phase 6 – Final Member checking*- Following the consensus meeting, the final results were distributed for comment and agreement. This also involved the final ranking of items – the “Top 10” lists were ranked in order of importance. 

### Public and patient involvement

This research was led throughout by PPI members. The research funding application was developed in conjunction with The Alzheimer Society of Ireland and a member of their Dementia Research Advisory Team. On commencement of the research, the PPI Advisory Group was set up. This group was recruited from the target population (but not considered to be research participants) and advised on all aspects of the research process. The PPI group were involved in the adaptation and development of the questionnaire, advising on recruitment strategies, and working with the wider group to decide on the ranked needs and recommendations that were brought forward from the consensus process.

### Data collection tools

The original questionnaire, developed and administered by Fredriksen-Goldsen & Kim (2017), was adapted as appropriate to the Irish context. The survey was based on the National Health, Aging, and Sexuality/Gender Study ( Fredriksen-Goldsen & Kim, 2017) and adapted with PPI group. Guides to survey design and implementation were also followed as described by Thayer-Hart and colleagues (2010) and Oppenheim (2000).

The questionnaire, which was hosted on QuestionPro, consisted of a number of different section- Demographics, Community and Service-Use, including health and dementia-care services; LGBTQIA+ identity, and Discrimination. A total of forty-six items were included in the questionnaire and both full and partially completed questionnaires were accepted.

The questionnaire went through several rounds of revisions after consulting with the PPI group. Before the final version of the questionnaire was sent for data collection, it was piloted with a small sample of people not included in the main study. The purpose of the pilot study was to test run the data collection method, identify potential issues, and examine its viability. A number of small edits and clarifications were made at this stage, but nothing that changed the overall content. A copy of the questionnaire can be found in the Extended data.

Online interviews and the consensus meeting were facilitated by using a secure Zoom account. The online interviews were audio-recorded. The consensus event was not recorded but notes were kept during the meeting (by MHO) and a record of decisions was kept.

### Participants

Participants were eligible to participate if they:

- Identified as a member of the LGBTQIA+ community or supported someone who is/was.
- Were aged over 50 years.
- Were able to provide informed consent.
- (Interview and consensus group only) were fluent in spoken English

People were ineligible to take part if they provided paid care for someone from the LGBTQIA+ community or were based outside of Ireland (exception for “international expert interviews”).

Participants were recruited through an email or social media post from gatekeepers at relevant organisations – e.g. The Alzheimer Society of Ireland, LGBT Ireland, TENI, Linc and other relevant local and national LGBTQIA+ organisations in Ireland. We used national LGBTQIA+ websites and magazines ( Gay Community News), and radio and television interviews ( TG4 and Radiό na Gaeltachta) to recruit participants. We also advertised through social media- Facebook and Twitter and paper versions of the questionnaire were available in a number of LGBTQIA+ community resource centres.

In order to consent to taking part in the survey, participants confirmed that they read the information sheet, and ticked boxes associated with the inclusion criteria to provide their consent. Participants did not need to reveal their name/signature in order to participate in the survey. Informed consent was obtained in writing for interview participants. The guidance on evaluation of capacity to consent from the British Psychological Society (2020) was followed. As such, obtaining consent was seen as a continuing process, not a one-off decision. It was unlikely that the person with dementia would lose capacity to consent during their involvement in the study, but their willingness to continue was checked regularly. Interview participants, with the exception of international experts, were invited to take part in the consensus meeting at recruitment stage and provided written consent to take part in the consensus meeting. Other consensus participants, representatives from voluntary and healthcare backgrounds, provided oral consent and written confirmation via email. The meeting was not recorded, and no personal information was collected during the consensus event- only a record of the scoring and ranking as a group was collected so oral consent was sufficient. 

Sampling stopped after no more responses were recorded in the survey for a period of two weeks. Anyone who expressed an interest in the taking part in an interview was interviewed. Expert interview sampling was guided by principles of data adequacy ( Levitt *et al*., 2017).

### Analysis

Data from the questionnaires was analysed descriptively and via content analysis. The responses were exported into an Excel file and screened for errors and omissions to ensure data integrity. Descriptive statistics were calculated, which include totals (n), percentages, as well as the means and standard deviations. Open-ended text was analysed using content analysis.

Qualitative data from interviews was analysed using reflexive thematic analysis. This was an iterative, recursive process. All interviews were audio-recorded and transcribed. All transcriptions were deidentified during the transcription process and audio-recordings were deleted immediately after transcription. Data were analysed as described by Braun and Clarke’s (2021) six-phase data analysis process. This entailed three main tasks: familiarisation with data- the transcripts were read and re-read and initial codes were developed; coding and theme identification- ongoing formation and revision of themes; and the reviewing and refining of themes. This facilitated an inductive approach to identifying, analysing and reporting the themes identified within the data collected ( Braun & Clarke, 2012). The first author was responsible for data analysis of both the survey and interview data, and verification of data integrity was conducted by the second author.

Consensus on a topic was decided if a certain percentage of the votes fell within a prescribed range ( Miller, 2006). This range was set at 70% of consensus participants agreeing that an item was important. Each of the needs and recommendations were calculated and ranked as they were scored by participants – this was done by adding the total score for each item and dividing it by the number of overall votes ( McMillan *et al.*, 2016). This ranked score was used to identify the “Top 10” needs and recommendations and presented to participants for discussion. The second author was involved in the score calculation and the first author reviewed the scores and informed the consensus participants.

Credibility and trustworthiness of the data was ensured through a number of triangulation strategies. This included having multiple observer/observations/data collection methods, as well as including two data analysts. The researchers immersed themselves in the data to ensure rich descriptions. Working with PPI Advisory group increased the validity of findings and the likelihood of collecting data that was useful to the group under study. 

## Results

### Survey responses

***Participant demographics***. Forty-nine responses were recorded in the survey with a 46.94% completion rate. Participants were aged between 50 and 75 years old. 2.78% of survey participants were LGBTQIA+ people living with dementia, 13.89% were a care partner of an LGBTQIA+ person with dementia and 83.33% were LGBTQIA+ adults over the age of 50.

57.14% self-identified as female, 39.29% self-identified as male and 3.57% selected epicene. 82.14% of participants were cisgender, 14.29% were transgender and 3.5% were unsure.

40.7% of survey participants identified as gay, and 40.7% identified as lesbian. 3.7% identified as bisexual and 14.8% identified as queer ^1^.

**Figure 1.**
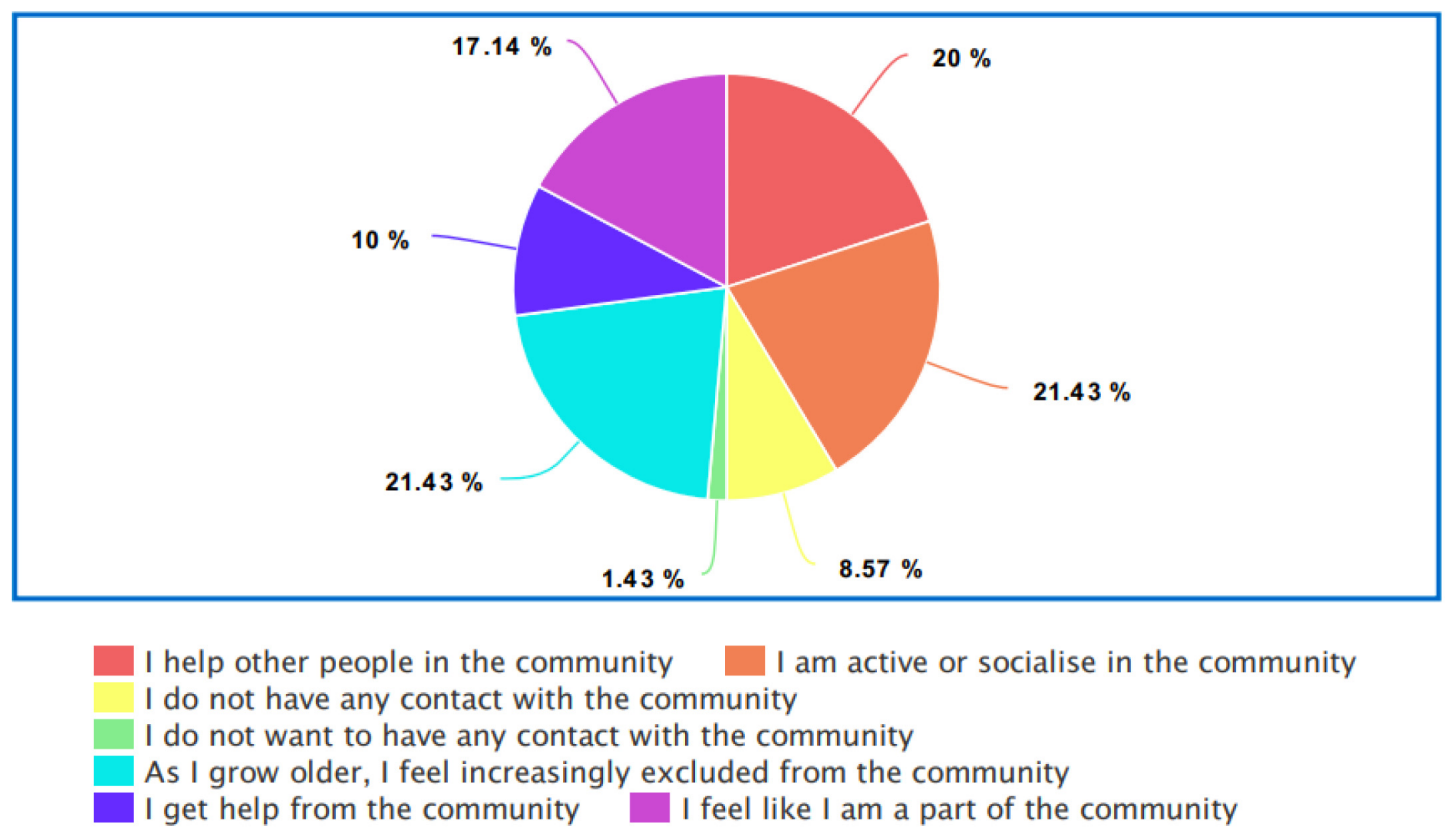
Participant community support.

***Community participation***. The data presented in Figure 1 indicates that there is a strong will and a need for socialization within the older LGBTQIA+ community. However, as 21.43% stated “As I grow older, I feel increasingly excluded from the community” and 8.57% stated that they have no contact with the LGBTQIA+ community, there is an indication that despite the general desire to be involved with the LGBTQIA+ community, older LGBTQIA+ adults between 50 and 75 years of age become more isolated from the LGBTQIA+ community.

Participants were asked “Prior to COVID-19, were you involved in any of the following activities?” and respondents answered Yes to a multitude of activities which are seen in Table 1.

**Table 1.** LGBTQIA+ Community Involvement (prior to COVID-19).

Community Activities	N	M	SD	Attending Activity %
Visited an LGBTQI+ pub or club	30	1.27	0.45	73.33
Attended an LGBTQI+ social group or outing	28	1.32	0.48	67.86
Attended or involved in an LGBTQI+ community event	29	1.31	0.47	68.97
Used a web-based LGBTQI+ discussion group/forum/dating site	30	1.50	0.51	50
Visited an LGBTQI+ community centre	28	1.46	0.51	53.57
Attended an LGBTQI+ support group	27	1.78	0.42	22.22
Other LGBTQI+ related activity or group	28	1.64	0.49	35.71

However, when asked about attendance of non-LGBTQIA+ specific services prior to COVID-19, fewer participants were in attendance. This is illustrated in Table 2. Only two activities were attended by participants in the majority of cases, these were LGBTQI+ groups and Cultural/arts groups.

**Table 2.** Non-LGBTQIA+ Community Involvement (prior to COVID-19).

Community Activities	N	M	SD	Attending Activity %
Residents’ Group	28	1.64	0.49	35.71
Sport Group	28	1.82	0.39	17.86
Religious Group	27	1.96	0.19	3.70
Political Group	27	1.67	0.48	33.33
Older person/ Active Retirement Group	27	1.67	0.32	11.11
Cultural/arts Group	28	1.46	0.32	53.57
LGBTQI+ Group	30	1.37	0.49	63.33
Other Group	23	1.74	0.45	26.09

When presented with a list of services and asked whether they would like LGBTQIA+-specific versions of those services, a large majority stated that they would like LGBTQIA+ specific versions of those services to be introduced, as seen in Table 3.

**Table 3.** Participant responses to need for LGBTQIA+ dementia services.

Service	N	M	SD	Answered “Yes”
Support groups/ memory café	28	1.14	0.36	85.71%
Social groups	27	1.04	0.19	96.3%
Reminiscence walking trails	26	1.08	0.27	92.31%
Community events/ social calendars	27	1	0	100%
Community Centre	27	1.07	0.27	92.59%
Support and befriending services	29	1.03	0.19	96.55%
Memories Choir	25	1.20	0.41	80%
Other	17	1.35	0.49	64.71%

Many of those who selected ‘other’ made suggestions for other LGBTQIA+-specific services, including an LGBTQIA+ medical support group or forum, an LGBTQIA+ retirement group, LGBTQIA+ specific support for people in the Traveller community, LGBTQIA+ care homes and residential living arrangements, and LGBTQIA+ specific ageing brain support.

**Figure 2.**
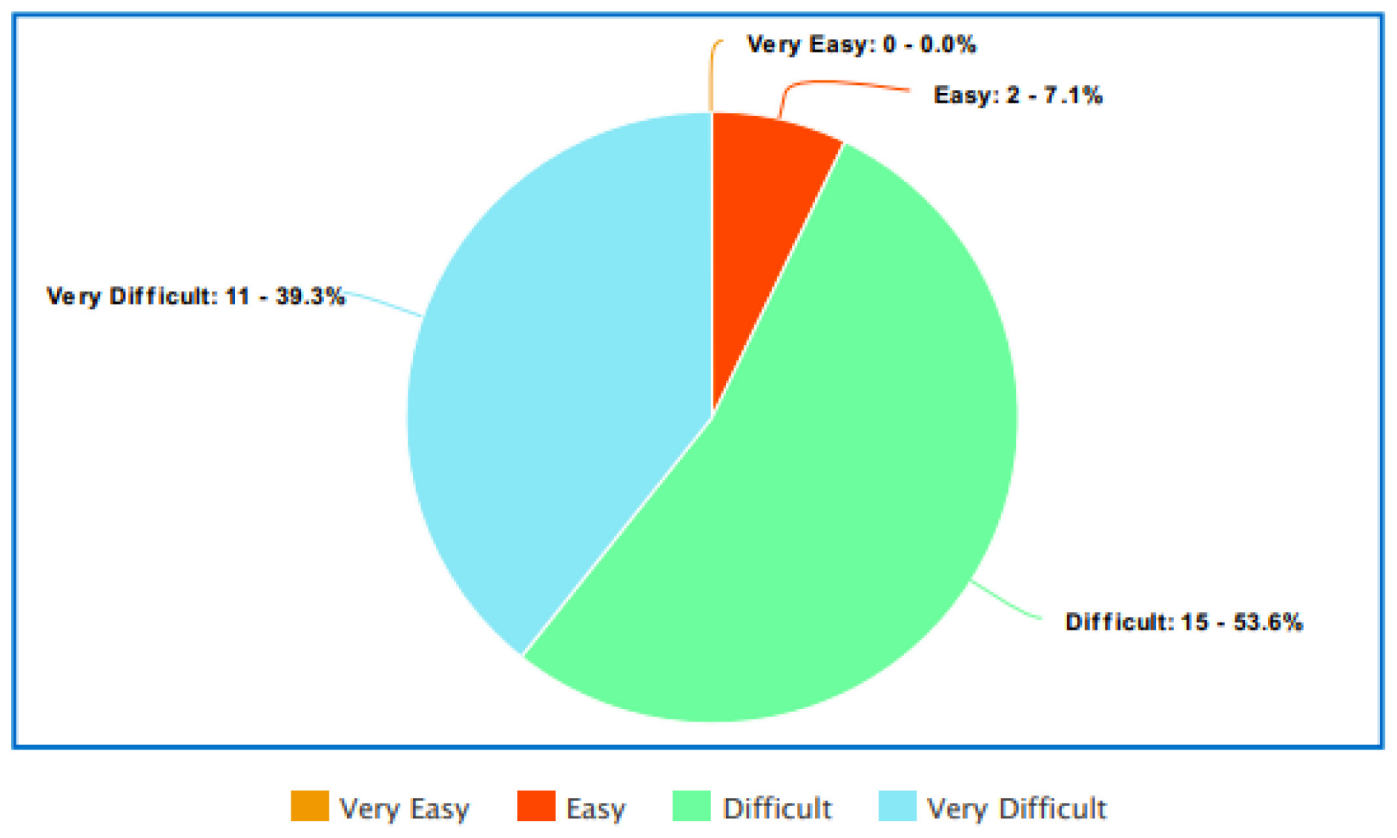
How easy is it to obtain LGBTQIA+ healthcare information?

***Healthcare access***. The majority of participants found it easy to access health information except in the instances where that health information was LGBTQIA+ specific. For example, the majority of participants declared that it was easy to find information on health issues that concern them, such as screening or regular health treatments, understand what their doctor said to them, judge the quality of health information from different sources, and to get the information they need from their doctor. However, when asked whether they found it easy to find health information from general sources that address the needs of LGBTQIA+ people, the majority of participants stated that this was difficult or very difficult, as illustrated by Figure 2 below.

**Figure 3.**
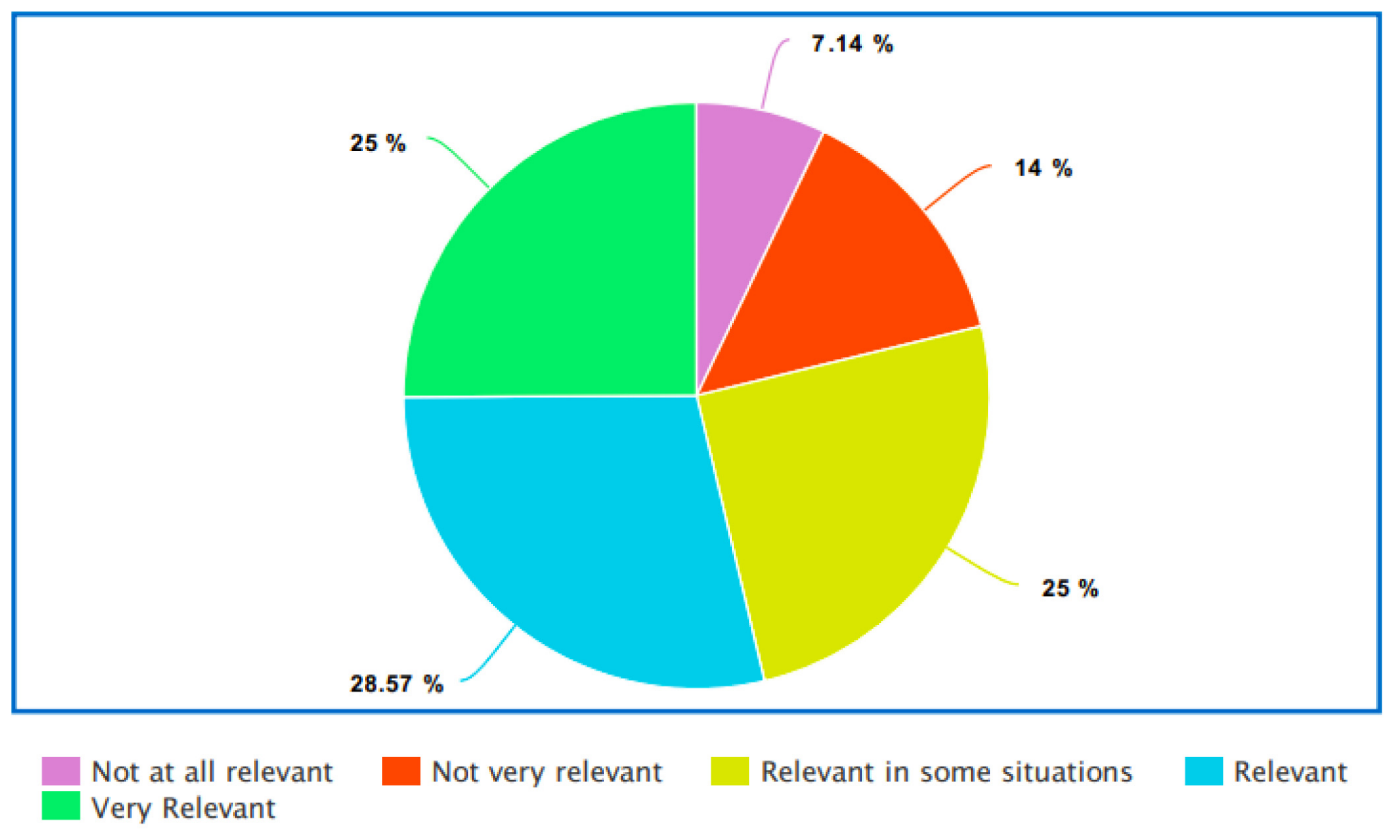
How relevant is LGBT identity to healthcare?

Participants were then asked how relevant their LGBTQIA+ identities were in a healthcare context. As illustrated in Figure 3, most participants believed that being LGBTQIA+ is relevant in a healthcare context.

The ethos of a care home also had the potential to contribute to anticipated disrespect. As one survey participant stated:

“ *As most care facilities are run by religious charities, there is a higher-than-average possibility that a person could encounter homophobia*”.

Like in prior research on this population, we found that many LGBTQIA+ older adults felt it necessary to conceal their gender/ sexual identity when accessing care/healthcare or stopped expressing their gender or sexual identity. As one survey participant stated:

“ *I fear many gay people are forced to pretend they’re straight as they get older and more isolated, just not to rock the boat*”.

Additionally, another survey participant stated,

“ *Well being gay is who I am. I have gay desires and sensitivities. They are part of my being, not some sort of aberration or problem. I think anyone who has to care for me should know this and act in a respectful way as a result. I should add that I have always had a positive experience in this regard to date*."

***Discrimination***. Participants reported that they have experienced multiple forms of abuse throughout their lifetime and within the past 5 years, as seen in Table 4.

**Table 4.** Lifetime abuse experienced by participants.

Type of abuse	N	M	SD	Lifetime %	Past 5 years %
Emotional abuse	16	1.88	0.34	87.50	12.50
Physical abuse	14	2	0	100.00	0.00
Verbal abuse	18	1.67	0.49	66.67	33.33
Sexual abuse	7	2	0	100.00	0.00
Psychological abuse	14	1.93	0.27	92.86	7.14
Racial abuse	3	1.67	0.58	66.67	33.33
Financial abuse	10	1.9	0.32	90.00	10.00
Organisational/Institutional abuse	11	1.82	0.4	81.82	18.18

An optional question surrounding the types of discrimination faced by participants was also included in order to gain a deeper understanding into where participants faced the most of their lifetime discrimination. As illustrated by Table 5, older LGBTQIA+ people have experienced discrimination in occupational, healthcare, and civil contexts.

**Table 5.** Discrimination experienced by participants.

	N	M	SD	Never %	Once %	Twice or more %
I was not hired for a job	20	1.85	0.99	55.00	5.00	40.00
I was not given promotion	19	2.00	0.94	42.11	15.79	42.11
I was fired from a job	16	1.56	0.89	68.75	6.25	25.00
I was prevented from living in the area I wanted	16	1.56	0.89	68.75	6.25	25.00
I was denied or provided inferior care such as healthcare	17	2.18	0.95	35.29	11.76	52.94
I felt unable to be open about my identity	19	2.68	0.67	10.53	10.53	78.95
My property was damaged or destroyed	17	1.71	0.92	58.82	11.76	29.41
I was hassled by the police	16	1.56	0.89	68.75	6.25	25

Participants also reported some day-to-day discrimination that they have experienced. For example, 39.13% participants reported that people do things to humiliate and devalue them a few times per year. 39.13% also reported that people suggest that they are inferior to others a few times per year and 39.13% report that they are treated with less curtesy and respect than others, a few times per year. However, the majority of participants never receive poorer service in shops or restaurants, are never made to feel less intelligent than others and have never had someone threaten to ‘out’ them to someone who they did not wish to disclose their identity to.

When asked why they experience discrimination several reasons were given by participants. One participant cited the lack of hate crime legislation in Ireland as causing a lack of protection form discrimination. Some participants mentioned that they were new to the community they were living in, others cited ageism, and many simply cited the fact that they were a gender or sexual minority.

Microaggressions were also experienced to some degree by participants. For example, 50% reported that people were dismissive of their “alternative” family structures and stable relationship and 54.55% of participants experienced negative stereotypes, a few times per year. However, between 13.64% and 31.82% did not experience the microaggressions listed in the survey.

Participants also displayed strong resilience in adverse situations, with 54.55% agreeing with the statement “I tend to bounce back quickly after hard times.”. 30.43% of participants also agreed with the statement “I usually come through difficult times with little trouble.”, with 21.74% strongly agreeing.

***Identity management***. All of participants in the survey have disclosed their sexuality or gender identity at least once, and the majority have disclosed this this more than once. 42.86% agreed that they are open about their sexuality whenever it comes up, and 38.10% strongly agreed. 40.91% agreed when they were assumed to be heterosexual/cisgender that they would correct them and 22.73% strongly agreed.

Participants emphasized the need to understand and respect the variety of networks and life experiences of LGBTQIA+ people. Many people noted that they are often assumed to be heterosexual or cisgender until told otherwise which can cause discomfort and puts the responsibility of disclosing gender and sexuality on the service user. As a survey participant stated:

“ *I'm okay with straight people caring for me and assume they would be tolerant - but the structures assume a heteronormative life*”.

61.9% strongly disagreed with the statement “I make things up to hide my sexual orientation or gender identity”, further suggesting that older LGBTQIA+ adults are open about their sexuality. A majority of participants also indicated that they display objects in their homes to suggest their sexual orientation or gender identity, which may be relevant for care providers entering the home.

**Figure 4.**
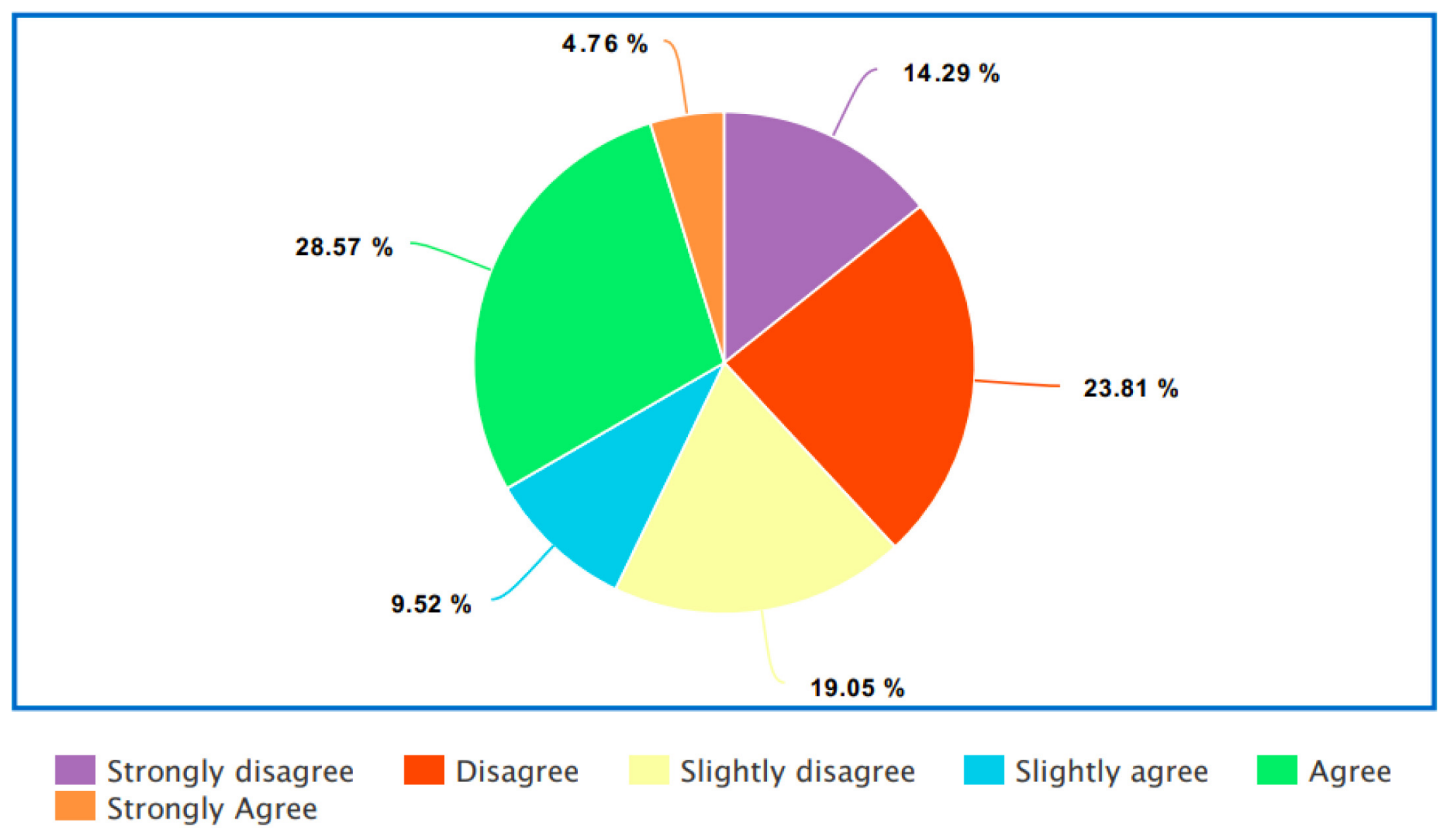
I have to work harder for my concerns to be heard and acted upon by health professionals where my LGBTQI+ identity is known.

42.86% strongly disagreed with the statement “I feel uncomfortable dealing with health professionals and official organizations where my LGBTQI+ identity is known”. However, different experiences were expressed with regards to the statement “I have to work harder for my concerns to be heard and acted upon by health professionals where my LGBTQI+ identity is known.”- see Figure 4.

Because of anticipated fears experienced by LGBTQIA+ older adults with regards to entering a care facility, it was suggested by numerous participants that a service that is LGBTQIA+ positive should display visual signs of acceptance, such as badges, flags, symbols, leaflets or pictures of same sex couples, however this should not be done without the adequate training of staff members.

Multiple participants also suggested the creation of an LGBTQIA+ accepting environment. LGBTQIA+ dedicated dementia services that reflect the heterogenous needs of the LGBTQIA+ community need to be created. For instance, one participant stated that:

“ *Ireland should have purpose-built residential care for LGBTQI+ older adults like they have in most other EU states and North America*.”.

Some participants did not like the idea of differentiated services and would prefer to be in a mixed setting. As a survey participant stated:

“ *I would like inclusive good quality services available generally in Ireland, not ghettoised services if no service is up to standard*.”

Another survey participant similarly stated:

*“Having visited my mother in a nursing home in Ireland for over 6 years I would rather die than enter one, especially without the prospect of an advocate of any type. Maybe that would be the answer, having an advocate”.*

The suggestion of having an advocate assigned to people was also discussed in the interviews. The findings of the interviews will be discussed now.

### Interview data

Inductive thematic analysis was performed on the six interviews conducted with international experts and LGBTQIA+ people over the age of 50. Two main themes were derived from these data:

1. Identity suppression and anticipated concerns
2. Creating an LGBTQIA+ affirmative ethos and workforce

***Identity suppression and anticipated concerns***. Many participants reported that they anticipated some forms of disrespect, such as homophobia, transphobia, humiliation, rudeness or isolation, if they were to enter a nursing home or become dependent on formal care. A transgender interview participant cited prior negative healthcare experiences as influencing his concern that he may be mistreated and humiliated in a care context. He stated:

“ *My biggest concern would be care staff, if I become very care dependent, would be to have my body mocked, or to be alienated, or that they would get sloppy with my medication regime. I'm honestly not even sure what medical recommendations would be about hormone treatment in high old age, because again our cohort are sort of a natural experiment…this kind of neglect of our particular situation*.”

An international expert stated that she would “ *rather die than be cared for in this place*” when she visited a care home with a very large crucifix on the wall.

“ *We hear stories of people being told you know ‘it’s not too late’ and being given bibles and being prayed over.*”

This suggests that even if a particular religious run service is LGBTQIA+ inclusive, the religious ethos alone may deter LGBTQIA+ service users from making use of it.

Interestingly a transgender interview participant cited very positive surgical and person-centred care experiences in a German hospital run by a Lutheran charity and a desire to utilise other religious care facilities in the future. This indicates, that though research has shown that religious ethos can have a direct negative impact on LGBTQIA+ service users, that this is not always the case when the care provided is person-centred at its core. He shared that he had very positive surgical experiences in Germany, but very poor experiences in Ireland, which has caused him to worry about what will happen when he is older and unable to challenge mistreatment.

“ *It was amazing apart from having a very good surgeon, the whole care staff, the house keeping staff, everyone was really, really affirming it was wonderful experience, and that makes it so hard to come back here be on your own and be raging at the national gender service because you have just been treated like a human and now you are back to just being a nuisance and left to fend for yourself*.”

Hearing and reading about accounts of older LGBTQIA+ people in care and suppressing their identity in care due to fears of social exclusion, discrimination, abuse or even differential treatment, can also contribute to older LGBTQIA+ people anticipating their own identity suppression. As one interview participant stated:

“ *I've been reading too many reports of cisgender gay and lesbian people who were forced to hide their sexuality in care settings, that’s all in the United States, but I think oh gee how will it turn out when it’s my turn?*”

High staff turnover was also considered a contributing factor to identity suppression and anticipated disrespect as it reduces consistency in care and can increase concerns over acceptance when trying to express one’s identity. An international expert participant discussed an instance in which she interviewed a lesbian woman in her 80’s who only first disclosed her sexuality to the manager in her nursing home, following her husband’s death:

*“[The manager] was very supportive and found her a local lesbian group...but the manager left and the next one she didn’t like and she’s now very frightened because she doesn’t know who knows and doesn’t know what people might say*.”

Finally, the inability to conceal was reported to leave older LGBTQIA+ people with dementia vulnerable, particularly in potentially unfriendly environments, confirming findings from previous studies. This was described by an older participant from LGBTQIA+ community:

*“...But for people who are old now, things can be revealed that are then held against them, which they’ve been able to manage, and the secrets they’ve been able to manage for all that time and the illness then robs them of that. Often, in addition guests and visitors, if your guests and visitors you know they could betray a sexual orientation as well so if you’re a gay man and you are 80 and the only people who come and visit you are men, you know people are going to draw conclusions...*”

***Additional note on transgender experiences and identity***. Unlike with the experiences of sexual minorities, trans identity is often overfocused on in a healthcare context, which can both waste a service-user’s time when trying to focus on non-transgender related health issues and can feel uncomfortable and unnecessary.

“ *I sometimes don’t like how I am outed by default, by all sorts of specialists who don’t really need to know…why would they need to know, and I am uncomfortable so it’s more the opposite, I am not so happy about every care provider in whatever remote context knowing I’m trans, you know sometimes I think it’s not necessary*.”

With regards to transgender dementia care, an international expert noted that there are two opposing schools of thought about gender affirmation in dementia. One school of thought, which the participant was opposed to, was to rigidly affirm the gender as expressed by the transgender person, before their diagnosis with dementia. This was opposed by the participant as although she acknowledged that many transgender people have had to fight for the duration of their lives for gender recognition, and do not wish to lose this, in many reported cases, transgender people with dementia can experience gender dysphoria and different gender identities can become more salient at different times, which can be confusing and distressing. She believed that to address someone as their previously preferred gender identity, whilst they are presenting or feeling like another would be “ *well-meaning coercion, but coercion none the less*”. Instead, this participant suggested the second school of thought, which is a more person-centred “ *take me as I am*” approach, in which care providers address a person as the gender that they are most comfortable with in that moment.

***Creating an LGBTQIA+ affirmative ethos and workforce***. Creating an explicitly LGBTQIA+ affirmative workforce was seen as paramount in creating safer and more welcoming care environments for LGBTQIA+ people with dementia, and a number of participants suggested that in order to create this affirming workforce, a number of steps needed to be set in place, including specific recruitment techniques, training, the use of visual signs of acceptance and the creation of dedicated services.

One participant noted the visibility of same sex couples of all ages in a Canadian LGBTQIA+ health care center was very positive.

“ *It was like I had died and gone to heaven surrounded by images of LGBTIQ people of all ages and same sex couples and also people who were gender fluid and exploring, it was just glorious*”.

In order to ensure that all new members of staff are LGBTQIA+ positive, or at least are open to being trained in LGBTQIA+ affirmation in care, it was suggested that care services advertise an explicit pro-LGBTQIA+ ethos. As one interview participant stated:

*“Now on their website there is a proactive, ‘we don’t tolerate any kind of discrimination, we are fully inclusive we welcome LGBTQ people’, I mean its screaming, ‘don't work for us if you don’t like LGBTQ people’ because we do. Then on its recruitment page it’s got the same message again, you can’t miss it its stark and, on its application, forms it says ‘do you understand this? Are you willing to commit to this?” So if anybody… really doesn’t like working with LGBTQ people, they’re not going to apply to [service name]. Not unless they’ve got their fingers in their ears, and their eyes closed and they’re going La La La La”*

Some participants suggested the need to diversify the workforce by hiring more LGBTQIA+ care workers, who would know how to socialise with other LGBTQIA+ people. Other participants, however, stated that the identity of the care provider was not so relevant as their dedication and level of training. Mandatory and comprehensive training was suggested by multiple participants, as people who were more biased towards LGBTQIA+ people would most likely skip the training if it were not mandatory. It was also suggested by participants that training should reflect the understanding of identities as opposed to just sexual behaviours, along with an understanding of the effects of attitudes and biases. For instance, an interview participant stated:

“ *I am concerned about an apparent absence of training specifically built into medical, nursing, and social care training in relation to sexuality and its impacts on older people because the attitudes towards older people are generally very poor in this country*.”

When the topic of dedicated services was discussed, participants had differing views on this. An interview participant stated that though he had never considered the idea of an LGBTQIA+ specific service, that he was very interested in the idea.

*“…let’s say I know of an LGBTQI care facility I would probably try to get into it to be honest, it would feel very enticing to be with your own people, also that way you have people with whom you can talk. I do know that current seniors LGBTQ seniors in care facilities is that they find it very isolating, the heteronormativity of their peers. How do you have conversations...folks like us...today’s young people are so much more integrated… In our generation you lived such a life apart in many ways and it would be so nice to live with people who know what that is.”*

Some participants did not like the idea of differentiated services and would prefer to be in a mixed setting. An international expert also pointed out that in many cases different demographics within the LGBTQIA+ community often desire different things, for instance, gay men typically preferred the idea of LGBTQIA+ specific services, whereas lesbian women, particularly those who live separatist lives, preferred women-only services, which highlights the need for choice in care for LGBTQIA+ older adults, as well as services that recognise the different needs within the LGBTQIA+ community. In some instances, it may be necessary to take part in sensitivity work with service users to prevent transgressions against people with more marginalized identities.

There is a need for non-nuclear family structures and friend networks that are more relied upon by LGBTQIA+ people, to be respected and understood by professional dementia care providers, as in some instances, people in the LGBTQIA+ community experience alienation from their family of origin, decreasing their overall support system. This can be exacerbated if the LGBTQIA+ person has also migrated, particularly if leaving behind a country where LGBTQIA+ identities are more severely discriminated against. LGBTQIA+ people are also less likely to have children, decreasing their likeliness of having an intergenerational cohort. This is not always the case, as many LGBTQIA+ people do have children, adopt, or have younger relatives or friends, indicating that it is also important to avoid assumptions and ask questions about a person’s available network. It was suggested that an independent advocate should be triggered upon a dementia diagnosis, who could act in an older person’s best interests in cases where an individual’s social network was smaller, or in cases where unaccepting families of origin, or other potentially exploitative people are acting against the best interest of the service user.

### Consensus process

Following the analysis of the interview and survey data, a consensus meeting was held with ten key stakeholders, who consisted of LGBTQIA+ people with dementia, LGBTQIA+ older adults, former caregivers of LGBTQIA+ older adults, and people who have worked with LGBTQIA+ older adults and/or people with dementia. The findings were presented in an oral presentation and the core needs and recommendations that came from the research were presented and clarified. There were ten core needs and sixteen recommendations derived from the data. Following the discussion, participants were asked to add any new needs or recommendations that they thought would be appropriate. These additional needs and recommendations were added to the full list. The complete unranked list of needs and the complete unranked list of recommendations can be seen in Table 6.

**Table 6.** Full list of unranked needs and recommendations.

*Full list of needs identified (not ranked)*	*Full list of recommendations identified (not ranked)*
Care that values your needs as individuals and as LGBTQI or A+ people.	LGBTQIA+ specific services for older adults and people with dementia should be introduced (i.e. support groups, Alzheimer’s cafés, day care, housing communities and nursing homes).
To feel safe in expressing your identity if you want to.	Integrated services with mandatory comprehensive training for staff should be available where dedicated services are unavailable.
Not to feel pressured into expressing your identity if you don’t want to/ or don’t feel safe.	LGBTQIA+ older adults should have a choice between integrated and dedicated services.
To be safe from abusive families of origin (if you have an abusive family of origin)	An explicitly LGBTQIA+ inclusive ethos message should be visible. Visible displays of LGBTQIA+ acceptance (such as pictures of same sex older adult couples, rainbow symbols and explicit LGBTQ-inclusivity statements) should be clearly displayed in leaflets and web-pages of dementia services. *This must be accompanied by an inclusive ethos and staff trained in LGBTQIA+ * *affirmative care.*
To be actively aided in socialization of all kinds, including LGBTQIA+ community socialization and hobbies (family alienation & social isolation are more common).	When working with transgender people with dementia, care providers should address them as the gender they are presenting as in the current moment and not engage in any kind of coercion regarding their gender expression.
To feel respected and for your partner to feel respected.	Care providers should understand and be sensitive to the fact that there are differences in the life experiences and needs within the LGBTQIA+ community.
To know that you, or your partner, are entering into a safe environment.	Service-users should be asked who they are closest to, and who they would like to help them in their care and decision making as their dementia symptoms progress, as opposed to assuming that the service user would want their families of origin, spouses or their children to be in charge of their care process.
To have dignity in all areas of treatment, especially end of life care.	Religious ethos should not be prioritized over a person-centered approach, inclusivity or acceptance.
Not to be treated as heterosexual until told otherwise.	Low-paid care home staff/ care providers should be paid more to reduce turnover and increase safety and consistency for service-users.
More care options.	Recruit more LGBTQIA+ staff.
In a nursing home/ residential care setting, to be safe from homophobic/transphobic bullying/mistreatment from other residents. ***(Added at consensus stage)***	Dementia organizations should actively work alongside LGBTQIA+ organizations to engage in research and the formulation of best practice for caring for LGBTQIA+ people with dementia.- e.g., best practice for caring for transgender people with dementia.
The need to support trans people with dementia while also recognising the reality of biology and that some supports may require a focus on sex and not gender. ***(Added at consensus stage)***	An optional section about sexuality and gender identity (i.e., preferred pronouns) should be included on healthcare forms.
Provide specific trans* and intersex medical training for doctors and care staff working with elderly LGBTQIA+ people, to enable them to work safely with unfamiliar bodies. ***(Added at consensus stage)***	At first contact with services/ at diagnosis, people with dementia or symptoms of cognitive decline should be given a multitude of resources including information about LGBTQIA+ services. (A subtle way of making sure everyone – even those in the closet receives this information without feeling as though an assumption has been made).
	Services’ LGBTQIA+ inclusiveness and training should be auditable by a relevant health authority.
	Independent advocates for people with dementia should be triggered upon diagnosis. Advocates can work with people with dementia and their close networks to give them the care they desire most.
	Training should include a focus on understanding differences in LGBTQIA+ networks and how to incorporate an individual’s network in care without making assumptions; as well as intervening with homophobic/transphobic bullying/mistreatment from family of origin/others.
	Celebrating LGBTQIA culture & identities through pride events. ***(Added at *** ***consensus stage)***
	Highlight diversity and that some groups may want to meet alone eg. Lesbian support group. ***(Added at consensus stage)***
	Health and well being of carers/caregivers to be considered and how to make them more sensitive to LGBTQ+ unique needs. ***(Added at consensus stage)***
	I suggest adding a recommendation on the importance of developing an implementation plan for the recommendations. ***(Added at consensus stage)***
	I think that there should not be any ‘religious exemption’ clause, to me a religious exemption is asking religious to be held to a lower moral standard than anyone else. ***(Added at consensus stage)***

Following the addition of the new needs and recommendations by the stakeholders, the voting took place. After this vote participants were presented with the ten recommendations and needs that achieved the highest scores. Participants were then asked to rank the top ten recommendations- which recommendations should be prioritised and in which order. The final top ten need and recommendations, along with the associated rank score are presented below.

***Top 10 needs identified***

1. To feel respected and for your partner to feel respected. (Score 9.6)
2. To feel safe in expressing your identity if you want to. (Score 9.5)
3. To know that you, or your partner, are entering into a safe environment. (Score 9.5)
4. To have dignity in all areas of treatment, especially end of life care. (Score 9.4)
5. Care that values your needs as individuals and as LGBTQI or A+ people. (Score 9.3)
6. To be safe from abusive families of origin (if you have an abusive family of origin). (Score 9.3)
7. In a nursing home/ residential care setting, to be safe from homophobic/transphobic bullying/mistreatment from other residents. (Score 9.1)
8. Not to feel pressured into expressing your identity if you don’t want to/ or don’t feel safe. Score (8.8)
9. Provide specific trans* and intersex medical training for doctors and care staff working with older LGBTQIA+ people, to enable them to work safely with unfamiliar bodies. (Score 8.8)
10. The need to support trans* people with dementia while also recognising the reality of biology and that some supports may require a focus on sex and not gender. (Score 8.3)
11. Not to be treated as heterosexual until told otherwise. (Score 7.5)
12. To be actively aided in socialisation of all kinds, including LGBTQIA+ community socialisation and hobbies (Score 7.3).
13. To have more care options. (Score 7.0)

***Top 10 recommendations***

1. At first contact with services/ at diagnosis, everyone should be given a multitude of resources including information about LGBTQIA+ services. (Score 7.25)
2. LGBTQIA+ older adults should have a choice between integrated and dedicated services. (Score 6.63)
3. Integrated services with mandatory comprehensive training for staff should be available where dedicated services are unavailable. (Score 6.38)
4. LGBTQIA+ specific services for older adults and people with dementia should be introduced. (Score 6.25)
5. Services’ LGBTQIA+ inclusiveness and training should be auditable by a relevant health authority. (Score 6.13)
6. Service-users should be asked who they would like to help them in their care and decision making as their dementia symptoms progress. (Score 6.0)
7. Independent advocates for people with dementia should be triggered upon diagnosis. Advocates can work with people with dementia and their close networks to give them the care they desire most. (Score 5.0)
8. Training should include understanding differences in LGBTQIA+ networks and how to incorporate an individual’s network in care without making assumptions; as well as intervening with homophobic/transphobic bullying/mistreatment from family of origin/other. (Score 4.25)
9. When working with transgender people with dementia, care providers should address them as the gender they are presenting as in the current moment and not engage in any kind of coercion regarding their gender expression. (Score 4.13)
10. An explicitly LGBTQIA+ inclusive ethos message and visible displays of LGBTQIA+ acceptance should be clearly displayed in leaflets and webpages of dementia services. This must be accompanied by staff trained in LGBTQIA+ affirmative care. (Score 3.0)

## Discussion

This research has identified a prioritised, consensus-developed, and PPI-driven list of needs and recommendations for healthcare delivery for people with dementia from the LGBTQIA+ community. Having developed this list, the next crucial step is the implementation of these findings into practice to ensure we are delivering a human rights-based care for people with dementia, as recommended by the World Health Organisation (2015). Throughout the work with PPI members and those who participated in the consensus process, there was stress placed on urgent need for the translation of this research into improved and more welcoming care for those from the LGBTQIA+ community.

The importance of maintaining identity came across in all phases of the research. For those living with dementia there is a duality in terms of managing dementia and also managing one’s own identity ( McParland & Camic, 2018). The conflict that people face in terms of who to disclose their identity to and in what context was evident in the findings, and echoes previous research describing the challenge of “giving yourself away vs. holding onto yourself” ( McParland & Camic, 2018). With a diagnosis of dementia, it can also be difficult for people to remember who they told what to, that can be distressing.

Respect was another clear message that came from the research data. As well as respect for identity, respecting families of choice and including them in care decisions, when appropriate, was apparent from the research findings. Previous research has referred to relationships for people with dementia from the LGBTQIA+ community as “sheltered harbours” where people feel safe and comfortable ( McParland & Camic, 2018). The focus on including family of choice in care decisions and plans came across clearly in this research. It can also be challenging for people to maintain healthcare regimes, such as long-term hormone therapy without assistance, and using the support systems that people already have in place has the potential to improve outcomes for people with dementia.

Safety when accessing services was also a priority for participants in this research. It is evident from previous research that avoidance of healthcare services can lead people to being admitted to residential care when it could have been avoided ( Westwood, 2016). The fear expressed by participants in becoming dependant on healthcare services because of possible neglect or mistreatment has been seen in earlier research ( Putney *et al.*, 2018). This deep-seated anxiety has been found to lead to identity concealment and chronic distress ( Putney *et al.*, 2018). Ensuring the people feel safe when accessing services should be fundamental. If older people from the LGBTQIA+ community do not feel safe (see the focus on safety in the “top 10” needs identified) this should be addressed immediately at a service-level.

There were conflicting views in relation to the need for dementia-specific services for LGBTQIA+ community. Even if not requested by all, participants agreed that it would be beneficial to have the choice to engage with these services as they are needed by some. The importance of welcoming, open, and non-judgemental services was identified as both a need and a recommendation. The importance of having an “explicit ethos” was discussed at length and the need for visual representation of all types of older people in services, including sub-groups such as older LGBTQIA+ people from the Travelling community. Linked with this was mandatory training for healthcare professionals and the need to integrate this at the beginning of career training. In a US cohort of paid dementia caregivers ( Nowaskie & Sewell, 2021), researchers found gaps in clinical preparedness and knowledge about LGBTQIA+ health care, especially in transgender care. The importance of this type of training being mandatory, integrated, and comprehensive was clear from the research data collected in phases 2-5 and has been reported elsewhere ( Fredriksen Goldsen *et al.*, 2014; Nowaskie & Sewell, 2021), particularly given the challenges often reported by trans and gender non-conforming people living in Ireland ( McNeil *et al.*, 2013).

### Limitations

This research had several limitations that require discussion. Firstly, the number of participants included in the research was small. This was anticipated by the research team and a number of steps were taken to ensure a consensus-based process- e.g the research contained multiple phases; the research was led by a representative PPI advisory group; the consensus meeting used purposive sampling to ensure representation across groups; and a number of recruitment avenues were used. 

The questionnaire itself was lengthy and required a level of concentration that may have unintentionally excluded those with more severe dementia. Although the research included incomplete questionnaires (46.94% completion rate) and allowed for the questionnaire to be completed by or with a caregiver, there are likely people who were unable to take part because of the this. The challenges posed by the COVID-19 pandemic limited the possibility of in-person data collection. Although the research team placed paper versions of the questionnaires in LGBTQIA+ community centres, many older people were sheltering at home and not attending these locations.

The questionnaire did not capture the views of caregivers (only 13.89% of the total sample) and it is suggested that further research looks at this cohort separately, as we know that this group often has fears about the future that are coloured by their own experiences of caregiving ( Price, 2011). We included caregivers in interview and consensus stages, but this is limited to a small number of caregivers.

As can be seen from the findings, all of the participants in the research have disclosed their gender or sexual identity to at least one other person. This indicates that there may be older people who have not disclosed to anyone at this point that have been excluded from the data.

Finally, the research team acknowledge that the term “older”, set for this research as 50+, will vary in terms of ethnicity and life expectancy due to health disparities. For instance, in 2016 only 3% of people from the Travelling community in Ireland were aged 65 or older and ageing in this community has been redefined as being aged 40+ as their life expectancy is 17% lower than the non-Travelling Irish community ( Gibney *et al.*, 2018). It may have been more prudent to set a lower age limit for people in the Travelling community to better capture their ageing experiences. Future researchers may also consider allowing participants to decide if they identify as “older” rather than having a cut-off for the research.

## Conclusion

Although older LGBTQIA+ adults demonstrate strong resilience ( Lyons *et al.*, 2013; Witten, 2014), many have significant worries about the future, particularly in the context of dementia care. This research has provided a clear list of needs and recommendations that have been identified by the older LGBTQIA+ community as urgent and essential for improving healthcare access, safety and quality of life in care. It is vital that the staff in healthcare, voluntary, and community services working with older people are trained in understanding the needs of LGBTQIA+ older adults with dementia, and that services are explicitly welcoming and respectful when supporting LGBTQIA+ people with dementia and their care partners.

## Data availability

### Underlying data

The data that support the findings of this study, including scores from the two rounds of voting, the questionnaire answers, and interview transcripts, are available on request from the corresponding author [S.M.H] with reasonable request. The interview transcripts of a participant who did not give consent for their transcripts to be shared will not be available. The dataset is not publicly available due to their containing information that could compromise the privacy of research participants. Due to the smaller sample of interview participants, and the specific and unique nature of some of the described life events of the participants that were paramount to analysis, de-identification is not sufficient to prevent possible recognition of the individuals.

### Extended data

Open Science Framework: Dementia service needs and recommendations for LGBTQIA+ community. https://doi.org/10.17605/OSF.IO/P3UJE ( Hynes, 2022).

This project contains the following extended data

- Paper version of National Survey (a copy of the questionnaire)
- Topic Guide (interviews with LGBTQIA+ older adults)
- Document (Interview guide with experts)
- Consensus Event Annotated Agenda 9 ^th^ December 2021 (Topic guide – consensus meeting)
- Are you LGBTQIA+ and aged 50 or over (Sample social media and physical recruitment poster)
- SRQR_Checklist_dementia (SRQR checklist)

Data are available under the terms of the Creative Commons Attribution 4.0 International license (CC-BY 4.0).

## Footnotes

^1^ A note on the use of the word “Queer”- At pilot stage we received feedback stating that the term Queer may be offensive to some and that it should be removed. The reasoning behind this feedback was that, although the term Queer has been reclaimed by the LGBTQI+ community, older LGBTQI+ adults may have experienced this as a slur throughout much of their lives and may still feel disenfranchised by the word. Following this, the research team discussed the issue with the PPI advisory group. In their feedback they noted that the term Queer is a flexible term that describes people who are in the LGBTQI+ community who do not fit into the “narrow” definitions that the other labels represent, and that people who use the term to describe themselves are often the most vulnerable in the LGBTQI+ community. They also agreed that in an Irish context the term could be considered hurtful to some people. As inclusivity and sensitivity were of upmost importance to the study, the decision was made, in conjunction with the PPI group, to keep the term Queer within the demographic ‘Sexuality’, with a disclaimer above stating that ‘Queer’ in the context of this study was meant to reflect LGBTQI+ identity it was by no means used to offend or cause hurt.
