# Developing consensus-based recommendations for the delivery of dementia services for the LGBTQIA+ community in the Republic of Ireland

**DOI:** 10.12688/hrbopenres.13505.2

**Published:** 2022-11-07

**Authors:** Megan H. Oglesby, Sinéad M. Hynes

**Affiliations:** 1 School of Health Sciences, National University of Ireland Galway, Galway, H91TK33, Ireland

**Keywords:** dementia, LGBTQIA+, older adults, healthcare access, healthcare recommendations, public and patient involvement

## Abstract

Background: The number of older LGBTQIA+ adults is set to rise significantly in the coming years. The rising numbers sit together with the rise in the number of people in Ireland diagnosed with dementia. In Ireland, no dementia-specific services exist for people from the LGBTQIA+ community. The aim of this research was to 1) identify the future needs that older LGBTQIA+ people and their care partners living in Ireland have in relation to dementia care service delivery; and to 2) develop consensus-based recommendations for dementia service provision in Ireland.

Methods: A six-phase consensus process was used to develop the lists of needs and recommendations: 1) development; 2) national survey; 3) interviews with key stakeholders; 4) international review of best practice; 5) consensus meeting; 6) final member checking. Participants, aged over 50, were based in Ireland, identified as a member of the LGBTQIA+ community or supported someone who is/was.

Results: Results are reported from the survey (n=49), individual interviews (n=8), and the consensus meeting (n=10). Participants have concerns related to identity management and suppression,creating an LGBTQIA+ affirmative ethos and workforce, and respect and safety. From the results and consensus process, a full list of ten prioritised needs and recommendations have been developed that focus specifically on dementia care in Ireland for the LGBTQIA+ community.

Conclusion: The older LGBTQIA+ community has identified essential priorities for improving healthcare access and safety. These priorities now need to be urgently implemented into clinical and dementia care services.

## Background

Many older people from the lesbian, gay, bisexual, transgender, queer/questioning, intersex, asexual + (LGBTQIA+) ^1^ community have experienced discrimination and marginalisation in their lives. As homosexuality was not decriminalised in Ireland until 24 June 1993, ( Criminal Law [Sexual Offences] Act, 1993) many of the older LGBTQIA+ people living in Ireland came of age at a time when same-sex behaviour or gender non-conformity was severely stigmatised and criminalised. Countless people left Ireland or concealed their gender and/or sexual identity because they felt uncomfortable or unsafe. Many older LGBTQIA+ people feel increasingly vulnerable as they age and have significant worries related to preparation for ageing, in particular trans and gender non-conforming older adults ( Sharek *et al.*, 2015). This is often compounded by previous negative life experiences.

The number of older LGBTQIA+ adults is set to rise significantly in the coming years, with the number of older people in general rising ( Sheehan & O’Sullivan, 2020) at the same time as more people are revealing their gender identity or sexual orientation later in life. Research has clearly shown that older LGTBQIA+ adults are less likely to engage withhealth services ( Higgins *et al.*, 2011) and community groups and are more likely to report poor general and mental health ( Fredriksen-Goldsen *et al*., 2015; Fredriksen-Goldsen & Kim, 2017; Wallace *et al.*, 2011). Some people articulate strong social support networks ( King & Cronin, 2016) but this is not the case for many ( Kim *et al.*, 2017), with increased levels of loneliness and isolation seen in this population ( Kuyper & Fokkema, 2010).

The number of people living with dementia in Ireland is also on the increase ( Alzheimer Europe, 2020). There is estimated to be between 39,272 and 55,266 people with dementia in Ireland, which is an increase of 7752 new cases per year ( Pierse *et al.*, 2019). There is also some debate as to whether older people from sexual minorities are at an elevated risk of cognitive impairment ( Perales-Puchalt *et al.*, 2019), with recent robust research suggesting that the rates of cognitive impairment appear to be significantly higher among sexual minority older adults than among heterosexual older adults, even when sociodemographic factors are adjusted for ( Hsieh *et al.*, 2021). This may be due to members of the LGBTQIA+ community being more at risk for conditions such as HIV and depression which in turn increase the likelihood of developing dementias ( Fredriksen-Goldsen *et al.*, 2015).

Health inequalities can be seen in this community and yet in Ireland very little, if anything, has been done to address the lack of diversity in health care delivery for older adults ( Roe *et al.*, 2020). Internationally examples of good practice in relation to dementia and older adult services for the LGBTQIA+ community exist, for example, the UK Government published National LGBT Action Plan in 2018 and appointed a National Advisor for LGBT Health which appears to be having a positive impact on health and well-being of the older LGBTQIA+ community ( Opening Doors London, 2021). However, at the time of conducting this study (2021), there were no dementia organizations in the Republic of Ireland that were offering any LGBTQIA+-specific service or advice. Nationally,there is an imperative need to ensure our health and care services are addressing the needs of under-served populations,such as vulnerable groups like older people from the LGBTQIA+ population. Building on recent recommendations in this area ( Roe *et al.*, 2020), this research aimed to:

1. Identify the future needs that older LGBTQIA+ people and their care partners living in Ireland have in relation to dementia care service delivery.
2. Develop consensus-based recommendations for dementia service provision in Ireland.

## Methods

Ethical approval for the research was granted by the National University of Ireland Galway Research Ethics Committee- Reference number 2021.05.010. Data collection commenced on July 2021 until December 2021. The Standards for Reporting Qualitative Research (SRQR; O’Brien *et al.*, 2014) were followed in reporting the results and the SRQR checklist can be found in the Extended Data.

### Design

The traditional Delphi consensus process (as described in Hsu & Sandford, 2007), which involves multiple iterations with highly trained and specialised Delphi participants, is not well suited for a population of people with dementia. The consensus process used here was adapted to ensure accessibility to people with dementia, older people, and care partners ( Morbey *et al.*, 2019). The research included older LGTBQIA+ people with and without dementia through the research process (with guidance from Swarbrick *et al.*, 2019). There was substantial member involvement throughout. A six-phase process took place in order to reach a consensus on recommendations and prioritised needs. 

### Public and patient involvement (PPI)

This research was led by PPI members. The research funding application was developed in conjunction with The Alzheimer Society of Ireland and a member of their Dementia Research Advisory Team. On commencement of the research, a PPI Advisory Group was set up. This group was recruited from the target population and advised on all aspects of the research process. The PPI group were involved in the adaptation and development of the questionnaire, advising on recruitment strategies, and working with the wider group to decide on the ranked needs and recommendations that were brought forward from the consensus process.

### Procedure

A six-phase consensus process was followed to identify the needs and recommendations, as described below.

***Phase One: Development.*** The questionnaire to be used was adapted as appropriate to the Irish context. The questionnaire was based on the National Health, Aging and Sexuality/Gender Study ( Fredriksen-Goldsen & Kim, 2017) from the US and adapted with the PPI group. The PPI group decided on the inclusion of specific items, length and format of the questionnaire, rating scales and accessibility of language. Guides to survey design and implementation were also followed as described by Thayer-Hart and colleagues (2010) and Oppenheim (2000).

The questionnaire was hosted on QuestionPro and consists of several different sections, including ‘Demographics’, ‘Community’ and ‘Service-Use’, ‘LGBTQI+ identity’, and ‘Discrimination’. A total of forty-six items were included in the questionnaire and both full and partially completed questionnaires were accepted. The questionnaire went through several rounds of revisions after consulting with the PPI group and was piloted on a small group of seven people prior to being used in the main study. Several small edits and clarifications were made at this stage, but nothing that changed the overall content. A copy of the questionnaire can be found in the Extended data linked at the end of the paper.

***Phase Two: National survey of older LGBTQI+ people and care partners.*** During Phase Two the questionnaire was distributed. Postal and online completion options were available to participants. Participants also had the option of completing the questionnaire over phone/video call. In order to consent to taking part in the survey, participants confirmed that they read the information sheet, and ticked boxes associated with the inclusion criteria to provide their consent. Data collection stopped and the survey was closed after no more responses were recorded in the survey for a period of two weeks.

Participants were eligible to participate if they:

- Identified as a member of the LGBTQI+ community or supported someone who is/was.
- Were aged over 50 years.
- Were able to provide informed consent.

People were not eligible to take part if they provided paid care or were based outside of Ireland.

Participants were recruited through an email or social media post from gatekeepers at relevant organisations – e.g. The Alzheimer Society of Ireland, LGBT Ireland, TENI, Linc and other relevant local and national LGBTQIA+ organisations in Ireland. We used national LGBTQIA+ websites and magazines ( Gay Community News), and radio and television interviews ( TG4 and Radiό na Gaeltachta) to recruit participants. We also advertised through social media, Facebook and Twitter,and paper versions of the questionnaire were available in a number of LGBTQIA+ community resource centres.

Data from the questionnaires were analysed descriptively and written answers were analysed via conceptual content analysis. The responses were exported into an Excel file and screened for errors and omissions to ensure data integrity. Descriptive statistics were calculated, which include totals (n), and percentages. Open ended text answers were read and re-read, initial codes were then developed that were reflective of the answers described within the data, and quantified in order to identify the pattern of core concepts as described by Elo & Kyngäs (2008).

***Phase Three: Interviews with key stakeholders.*** Older LGTBQI+ adults and care partners were interviewed via Zoom. The aim of the interview was to gain more in-depth information on needs that may not captured in the survey and to further discuss future care needs. Informed consent was obtained in writing for interview participants. The guidance on evaluation of capacity to consent from the British Psychological Society (2020) was followed. As such, obtaining consent was seen as a continuing process, not a one-off decision. Anyone who expressed an interest in taking part in an interview was interviewed. The online interviews were audio-recorded, and deleted following transcription. A copy of the topic guide can be found in the Extended data linked at the end of the paper.

Qualitative data from interviews (phase three and four) was analysed using reflexive thematic analysis. This was an iterative, recursive process. All interviews were audio-recorded and transcribed. All transcriptions were de-identified during the transcription process and audio-recordings were deleted immediately after transcription. Following this, the transcripts were read and re-read and initial codes were developed. From this there was ongoing formation and revision of themes, which facilitated an inductive approach to identifying, analysing and reporting the themes identified within the data collected ( Braun & Clarke, 2012).

Credibility and trustworthiness of the data was ensured through a number of triangulation strategies. This included having multiple data collection methods, as well as including two data analysts. The researchers immersed themselves in the data to ensure rich descriptions. Working with PPI Advisory group increased the validity of findings and the likelihood of collecting data that was useful to the group under study.

***Phase Four: International review of best practice.*** This phase involved a review of literature in the area, as well as policies and frameworks developed in other countries. Where a pre-existing technique/policy/framework was used internationally, but not in Ireland, the recommendation was included to be discussed and voted on by the consensus group.

As part of this phase, we also interviewed international experts (via Zoom) working or conducting research in dementia care with/for the LGBTQIA+ community. The aim was to develop a representation of what best practice in the area looked like internationally. Expert interview sampling was guided by principles of data adequacy ( Levitt *et al*., 2017). A copy of the topic guide can be found in the Extended data linked at the end of the paper.

***Phase Five: (Virtual) consensus meeting.*** The aim of the consensus meeting was to agree on a set of needs and recommendations. The consensus meeting involved the PPI advisory group (n=6), those who took part in the individual interviews (n=1), and representatives from voluntary and healthcare backgrounds (n=2) working with people with dementia (n=1). Ten key stakeholders, who consisted of LGBTQI+ people with dementia, LGBTQI+ older adults, former caregivers of LGBTQI+ older adults, and people who have worked with LGBTQI+ older adults and/or people with dementia took part in the consensus meeting. A purposeful sampling strategy was used to ensure a diverse group with varied experiences and backgrounds.

 Interview participants (not international experts) were invited to take part in the consensus meeting at the recruitment stage and provided written consent to take part in the consensus meeting. Other consensus participants, representatives from voluntary and healthcare backgrounds, provided oral consent and written confirmation via email. The meeting was not recorded, and no personal information was collected during the consensus event. Only a record of the scoring and ranking as a group was collected. Because of the virtual nature of the meeting and because participants may have been experiencing cognitive impairment, the number of participants included in the meeting was kept low (maximum 10).

The meeting used a modified nominal group technique to ensure the participation of all members and was guided by similar research in the area ( Brett *et al.*, 2017; Keegan *et al.*, 2021; Schneider *et al.*, 2016). The nominal group technique was used because it reduces the burden on participants and results can be obtained quickly and presented back to the group ( McMillan *et al.*, 2016). The following process was followed (a copy of the annotated agenda can be found in the Extended data linked at the end of the paper):

- Results of phases 2, 3, and 4 were presented to the group, along with the needs and recommendations that came from the research. The initial list of needs and recommendations were derived from the developed themes from the analysis of the survey, review of literature, interviews and long-answer survey responses.
- Sli.do, an online polling tool, was used to facilitate the adding, voting, and ranking of items.
- Completed silent generation when participants had the opportunity to think about any additional items they wanted to add;
- Completed a round robin where participants added those items anonymously
- Participants were provided with the opportunity to discuss any new items or seek clarification;
- Private voting and ranking of items took place- two rounds.
  - Round one- ranked importance of items.
  - Round two- ranked order of importance when “top 10” were identified.

Consensus on a topic was decided if a certain percentage of the votes fell within a prescribed range ( Miller, 2006). This range was set at 70% of consensus participants agreeing that an item was important. Each of the needs and recommendations were calculated and ranked as they were scored by participants – this was done by adding the total score for each item and dividing it by the number of overall votes ( McMillan *et al.*, 2016).

***Phase Six: Final Member checking.*** Following the consensus meeting, the results were distributed back to the consensus participants for comment and agreement. All participants agreed with the final list of items and their ranking.

## Results

### Quantitative Survey responses

***Participant demographics.*** Forty-nine responses were recorded in the survey with a 46.94% completion rate. Completion rate refers to the number of participants who completed the survey in its entirety. No postal survey responses were received. Participants were aged between 50 and 75 years old, mean age of 60.88 (SD 7.35). Table 1 contains the breakdown of participant demographic characteristics.

**Table 1.** Demographic characteristics of participants.

Participants self-identified as:	Percentage
LGBTQI+ adult over the age of 50 (without dementia)	83.33
LGBTQI+ adult over the age of 50 living with dementia.	13.89
Care partner of an LGBTQI+ person with dementia	2.78
***Gender Identity***	
Woman	57.14
Non-binary	0.00
Man	39.29
***Do you consider yourself:***	
Cisgender	82.14
Epicene	3.57
Transgender	14.29
Unsure	3.50
***Sexual Orientation***	
Gay	40.70
Lesbian	40.70
Bisexual	3.70
Queer	14.80
Heterosexual	0.00
Pansexual	0.00
Asexual	0.00
Questioning	0.00

**Figure 1.**
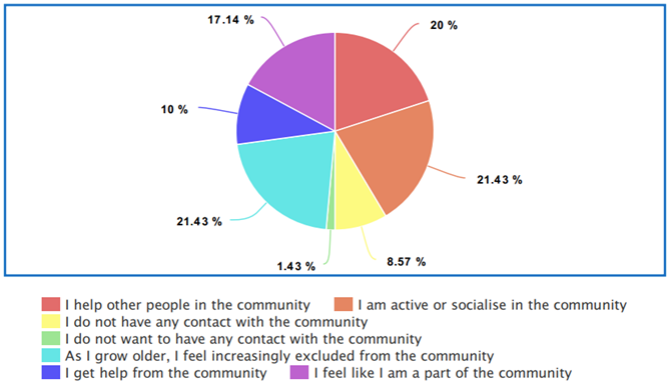
Participant community support.

***Community participation.*** The data presented in Figure 1 indicates that there is a strong will and a need for socialization within the older LGBTQI+ community. However, as 21.43% stated “As I grow older, I feel increasingly excluded from the community” and 8.57% stated that they have no contact with the LGBTQI+ community, there is an indication that despite the general desire to be involved with the LGBTQI+ community, older LGBTQI+ adults between 50 and 75 years of age become more isolated from the LGBTQI+ community.

Participantswere asked “Prior to COVID-19, were you involved in any of the following activities?” and respondents answered Yes to a multitude of activities which are seen in Table 2.

**Table 2.** LGBTQIA+ Community Involvement (prior to COVID-19).

Community Activities	N	Attending Activity %
Visited an LGBTQI+ pub or club	30	73.33
Attended an LGBTQI+ social group or outing	28	67.86
Attended or involved in an LGBTQI+ community event	29	68.97
Used a web-based LGBTQI+ discussion group/forum/dating site	30	50.00
Visited an LGBTQI+ community centre	28	53.57
Attended an LGBTQI+ support group	27	22.22
Other LGBTQI+ related activity or group	28	35.71

However, when asked about attendance of non-LGBTQI+ specific services prior to COVID-19, fewer participants were in attendance. This is illustrated in Table 3. Only two activities were attended by participants in the majority of cases, these were LGBTQI+ groups and Cultural/arts groups.

**Table 3.** Non-LGBTQIA+ Community Involvement (prior to COVID-19).

Community Activities	N	Attending Activity %
Residents’ Group	28	35.71
Sport Group	28	17.86
Religious Group	27	3.70
Political Group	27	33.33
Older person/ Active Retirement Group	27	11.11
Cultural/arts Group	28	53.57
LGBTQI+ Group	30	63.33
Other Group	23	26.09

When presented with a list of services and asked whether they would like LGBTQI+-specific versions of those services, a large majority stated that they would like LGBTQI+ specific versions of those services to be introduced, as seen in Table 4.

**Table 4.** Participant responses to need for LGBTQIA+ dementia services.

Service	N	Percentage Answered “Yes”
Support groups/ memory café	28	85.71
Social groups	27	96.30
Reminiscence walking trails	26	92.31
Community events/ social calendars	27	100.00
Community Centre	27	92.59
Support and befriending services	29	96.55
Memories Choir	25	80.00
Other	17	64.71

Many of those who selected ‘other’ made suggestions for other LGBTQI+-specific services, including an LGBTQI+ medical support group or forum, an LGBTQI+ retirement group, LGBTQI+ specific support for people in the Traveller community, LGBTQI+ care homes and residential living arrangements, and LGBTQI+ specific ageing brain support.

**Figure 2.**
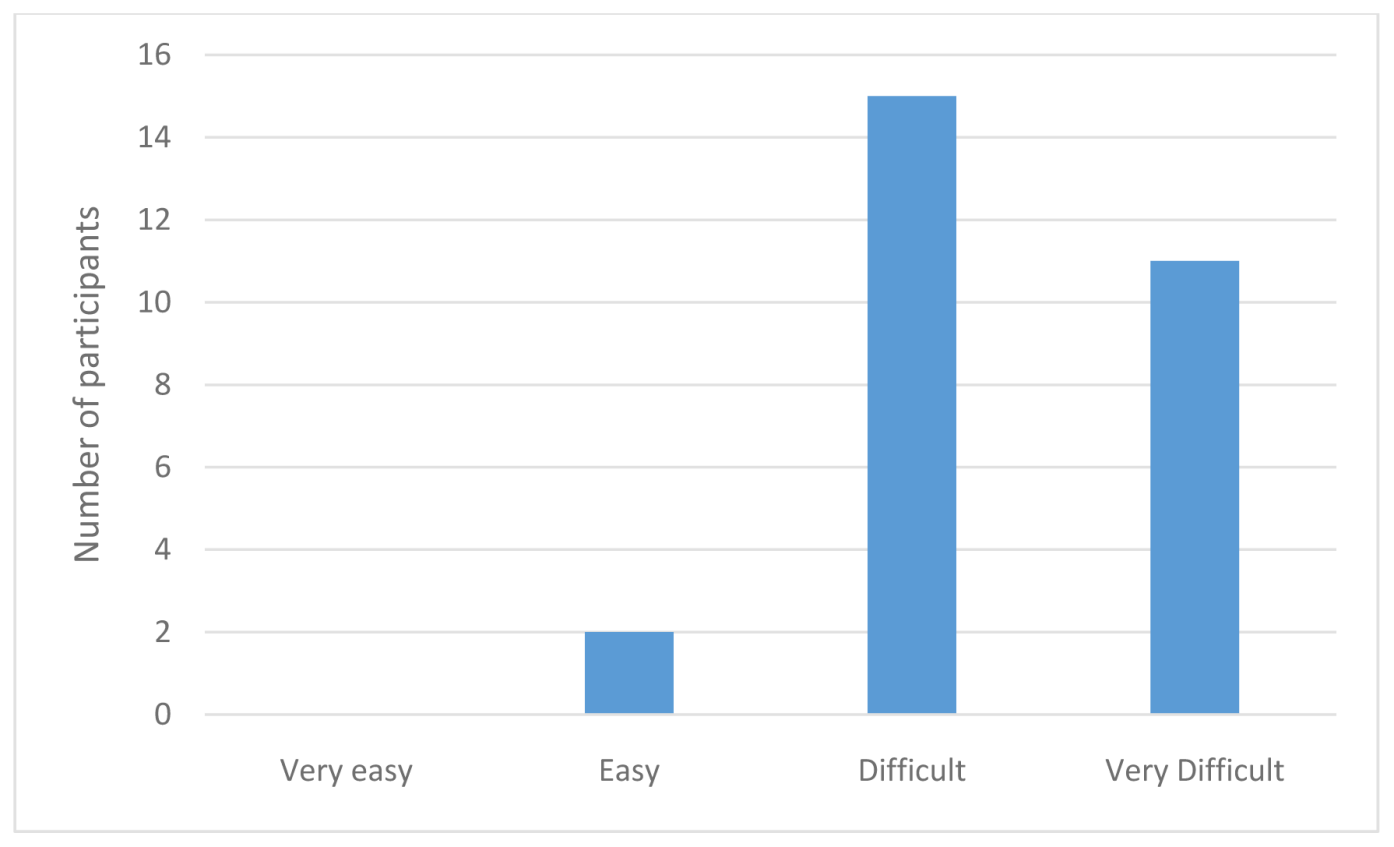
How easy is it to obtain LGBTQI+ healthcare information?

***Healthcare access.*** The majority of participants found it easy to access health information except in the instances where that health information was LGBTQI+ specific. For example, the majority of participants declared that it was easy to find information on health issues that concern them, such as screening or regular health treatments, understand what their doctor said to them, judge the quality of health information from different sources, and to get the information they need from their doctor. However, when asked whether they found it easy to find health information from general sources that address the needs of LGBTQI+ people, the majority of participants stated that this was difficult or very difficult, as illustrated by Figure 2 below.

**Figure 3.**
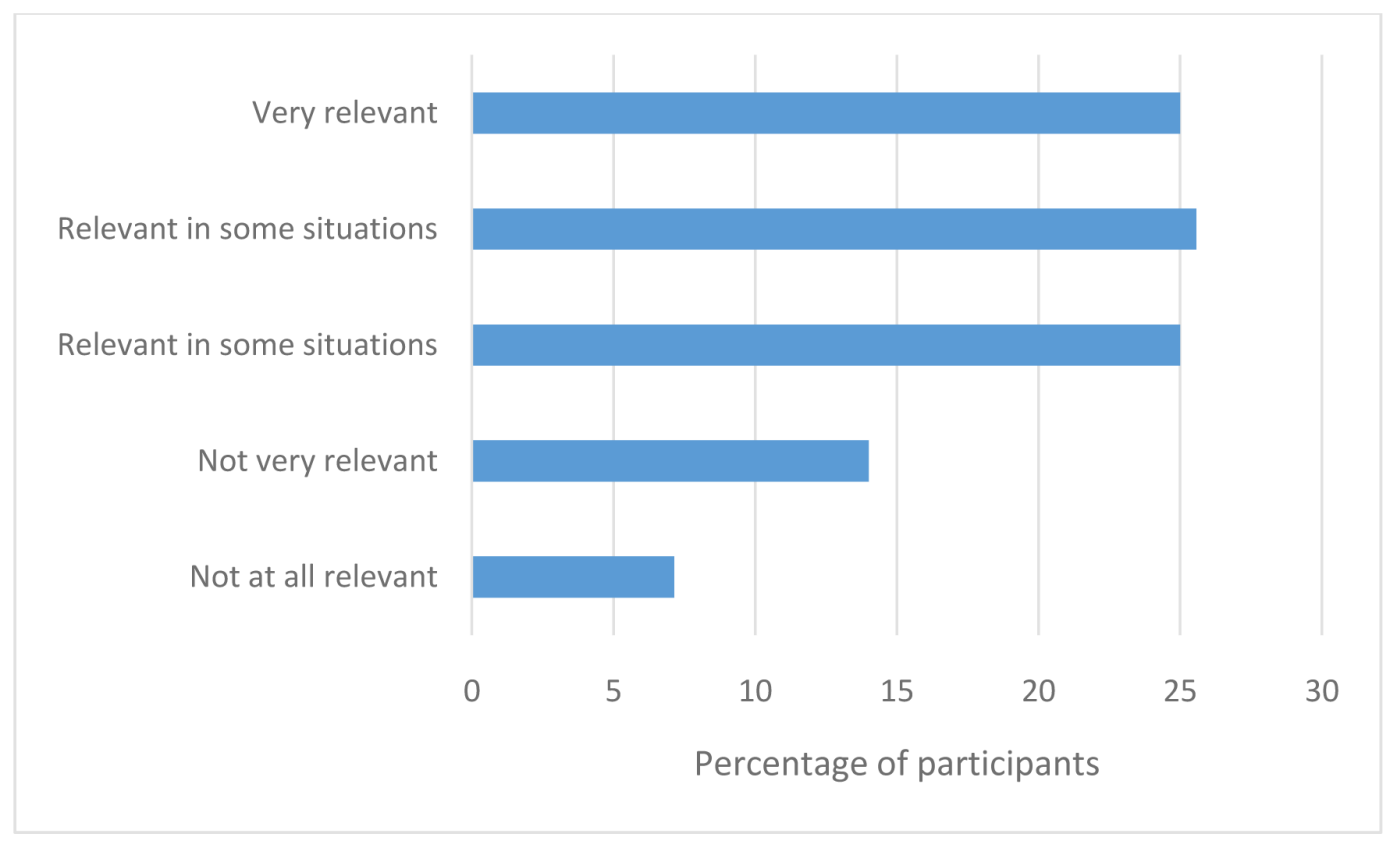
How relevant is LGBT identity to healthcare?

Participants were then asked how relevant their LGBTQI+ identities were in a healthcare context. As illustrated in Figure 3, most participants believed that being LGBTQI+ is relevant in a healthcare context.

***Discrimination.*** Participants reported that they have experienced multiple forms of abuse throughout their lifetime and within the past 5 years, as seen in Table 5. It is worth noting that 18 participants completed this question. Abuse was reported across all categories by participants during their life, with abuse in some categories being reported in the last five years- e.g. verbal abuse reported by 33%.

**Table 5.** Lifetime abuse experienced by participants.

Type of abuse	N	Lifetime %	Past 5 years %
Emotional abuse	16	87.50	12.50
Physical abuse	14	100.00	0.00
Verbal abuse	18	66.67	33.33
Sexual abuse	7	100.00	0.00
Psychological abuse	14	92.86	7.14
Racial abuse	3	66.67	33.33
Financial abuse	10	90.00	10.00
Organisational/Institutional abuse	11	81.82	18.18

An optional question surrounding the types of discrimination faced by participants was also included in order to gain a deeper understanding into where participants faced the most of their lifetime discrimination. As illustrated by Table 6, older LGBTQI+ people have experienced discrimination in occupational, healthcare, and civil contexts. Importantly, 78.95% of people have felt unable to be open about my identity twice or more.

**Table 6.** Discrimination experienced by participants.

	N	Never %	Once %	Twice or more %
I was not hired for a job	20	55.00	5.00	40.00
I was not given promotion	19	42.11	15.79	42.11
I was fired from a job	16	68.75	6.25	25.00
I was prevented from living in the area I wanted	16	68.75	6.25	25.00
I was denied or provided inferior care such as healthcare	17	35.29	11.76	52.94
I felt unable to be open about my identity	19	10.53	10.53	78.95
My property was damaged or destroyed	17	58.82	11.76	29.41
I was hassled by the police	16	68.75	6.25	25.00

Participants also reported some day-to-day discrimination that they have experienced. For example, 39.13% participants reported that people do things to humiliate and devalue them a few times per year. 39.13% also reported that people suggest that they are inferior to others a few times per year and 39.13% report that they are treated with less curtesy and respect than others, a few times per year. However, the majority of participants never receive poorer service in shops or restaurants, are never made to feel less intelligent than others and have never had someone threaten to ‘out’ them to someone who they did not wish to disclose their identity to.

When asked why they experience discrimination several reasons were given by participants. One participant cited the lack of hate crime legislation in Ireland as reason why people do not feel protected from discrimination in Ireland. causing a lack of protection form discrimination. Some participants mentioned that they were new to the community they were living in, others cited ageism, and many simply cited the fact that they were a gender or sexual minority.

Microaggressions were also experienced to some degree by participants. For example, 50% reported that people were dismissive of their “alternative” family structures and stable relationship and 54.55% of participants experienced negative stereotypes, a few times per year. However, between 13.64% and 31.82% did not experience the microaggressions listed in the survey.

Participants also displayed strong resilience in adverse situations, with 54.55% agreeing with the statement “I tend to bounce back quickly after hard times”. 30.43% of participants also agreed with the statement “I usually come through difficult times with little trouble”, with 21.74% strongly agreeing.

***Identity management.*** All of participants in the survey have disclosed their sexuality or gender identity at least once, and the majority have disclosed this this more than once. 42.86% agreed that they are open about their sexuality whenever it comes up, and 38.10% strongly agreed. 40.91% agreed when they were assumed to be heterosexual/cisgender that they would correct them and 22.73% strongly agreed.

61.9% strongly disagreed with the statement “I make things up to hide my sexual orientation or gender identity”, further suggesting that older LGBTQI+ adults are open about their sexuality. A majority of participants also indicated that they display objects in their homes to suggest their sexual orientation or gender identity, which may be relevant for care providers entering the home.

**Figure 4.**
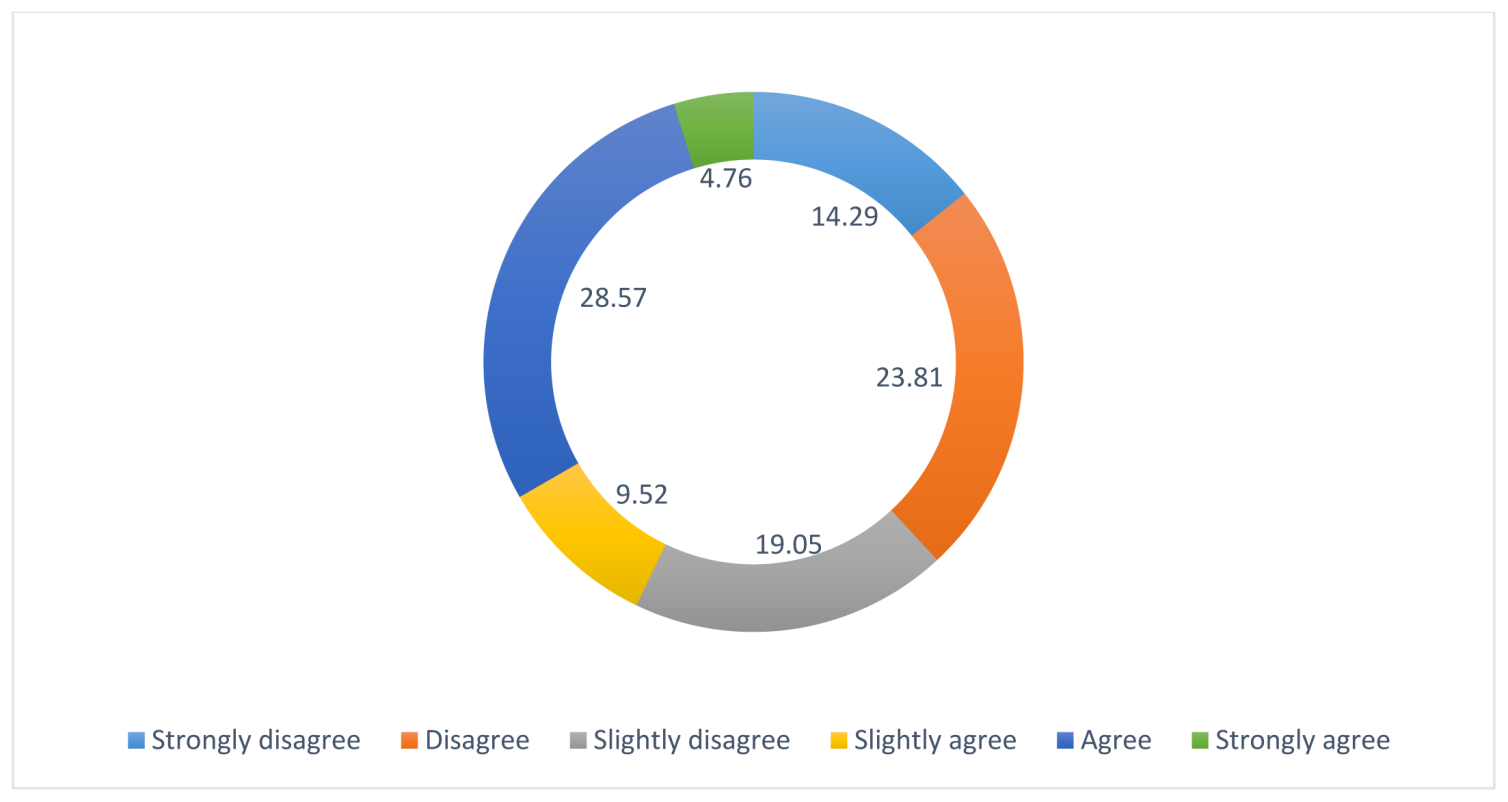
Percentage ranks for question: “ *I have to work harder for my concerns to be heard and acted upon by health professionals where my LGBTQI+ identity is known*”.

42.86% strongly disagreed with the statement “I feel uncomfortable dealing with health professionals and official organizations where my LGBTQI+ identity is known”. However, different experiences were expressed with regards to the statement “I have to work harder for my concerns to be heard and acted upon by health professionals where my LGBTQI+ identity is known.”- see Figure 4.

### Analysis of qualitative data- interviews and survey

Inductive thematic analysis was performed on the six interviews conducted with international experts and two LGBTQI+ people over the age of 50. Four main themes were derived from these data:

1. Identity suppression and anticipated concerns
2. Creating an LGBTQI+ affirmative ethos and workforce
3. Understanding the variety of LGBTQI+ networks and life experiences
4. Experiences specific to those who are transgender.

***Identity suppression and anticipated concerns.*** Many participants reported that they anticipated some forms of disrespect, such as homophobia, transphobia, humiliation, or isolation, if they were to enter a nursing home or become dependent on formal care. The ethos of a care home had the potential to contribute to anticipated disrespect. As one survey participant stated:

“ *As most care facilities are run by religious charities, there is a higher-than-average possibility that a person could encounter homophobia*”. Survey participant, lesbian cis-gender woman, 65 years.

We found that many LGBTQI+ older adults felt it necessary to conceal their gender/ sexual identity when accessing care/healthcare or stopped expressing their gender or sexual identity. As one survey participant stated:

“ *I fear many gay people are forced to pretend they’re straight as they get older and more isolated, just not to rock the boat*”. Survey participant, lesbian cis-gender woman, 57 years

Additionally, another survey participant stated,

“ *Well being gay is who I am. I have gay desires and sensitivities. They are part of my being, not some sort of aberration or problem. I think anyone who has to care for me should know this and act in a respectful way as a result. I should add that I have always had a positive experience in this regard to date*." Survey participant, lesbian cis-gender woman. 72 years

An international expert stated that she would “ *rather die than be cared for in this place*” when she visited a care home with a very large crucifix on the wall.

“ *We hear stories of people being told you know ‘it’s not too late’ and being given Bibles and being prayed over.*”Interview participant, international expert

This suggests that even if a particular religious-run service is LGBTQI+ inclusive, the religious ethos alone may deter LGBTQI+ service users from making use of it. Interestingly a transgender interview participant cited very positive surgical and person-centred care experiences in a German hospital run by a Lutheran charity. His positive surgical experiences in Germany, were followed by very poor experiences in Ireland, which has caused him to worry about what will happen when he is older and unable to challenge mistreatment.

“ *It was amazing apart from having a very good surgeon, the whole care staff, the house keeping staff, everyone was really, really affirming it was wonderful experience, and that makes it so hard to come back here be on your own and be raging at the national gender service because you have just been treated like a human and now you are back to just being a nuisance and left to fend for yourself*.” Interview participant, gay trans-gender man.

Hearing and reading about accounts of older LGBTQI+ people in care and suppressing their identity in care due to fears of social exclusion, discrimination, abuse or even differential treatment, can also contribute to older LGBTQI+ people anticipating their own identity suppression. As one interview participant stated:

“ *I've been reading too many reports of cisgender gay and lesbian people who were forced to hide their sexuality in care settings, that’s all in the United States, but I think oh gee how will it turn out when it’s my turn?*”Interview participant, gay trans-gender man.

High staff turnover was also considered a contributing factor to identity suppression and anticipated disrespect as it reduces consistency in care and can increase concerns over acceptance when trying to express one’s identity. An international expert participant discussed an instance in which she interviewed a lesbian woman in her 80’s who only first disclosed her sexuality to the manager in her nursing home, following her husband’s death:

*“[The manager] was very supportive and found her a local lesbian group...but the manager left and the next one she didn’t like and she’s now very frightened because she doesn’t know who knows and doesn’t know what people might say*.”Interview participant, international expert

Finally, the inability to conceal was reported to leave older LGBTQI+ people with dementia vulnerable, particularly in potentially unfriendly environments.

*“...But for people who are old now, things can be revealed that are then held against them, which they’ve been able to manage, and the secrets they’ve been able to manage for all that time and the illness then robs them of that.”* Interview participant, international expert

***Creating an LGBTQI+ affirmative ethos and workforce.*** Numerous participants suggested that a service that is LGBTQI+ positive should display visual signs of acceptance, such as badges, flags, symbols, leaflets or pictures of same sex couples. It was, however, emphasised that this should not be done without the adequate training of staff members. Multiple participants also suggested the creation of an LGBTQI+ accepting environment. LGBTQI+ dedicated dementia services that reflect the heterogenous needs of the LGBTQI+ community need to be created. For instance, one participant stated that:

“ *Ireland should have purpose-built residential care for LGBTQI+ older adults like they have in most other EU states and North America*.” Survey participant, gay epicene participant, 66 years

Some participants did not like the idea of differentiated services and would prefer to be in a mixed setting. As a survey participant stated:

“ *I would like inclusive good quality services available generally in Ireland, not ghettoised services if no service is up to standard*.” Survey participant, lesbian cis-gender woman. 64 years

Creating an explicitly LGBTQI+ affirmative workforce was seen as paramount in creating safer and more welcoming care environments for LGBTQI+ people with dementia, and a number of participants suggested that in order to create this affirming workforce, a number of steps needed to be set in place, including specific recruitment techniques, training, the use of visual signs of acceptance and the creation of dedicated services. One participant noted the visibility of same-sex couples of all ages in a Canadian LGBTQI+ healthcare center was very positive.

“ *It was like I had died and gone to heaven surrounded by images of LGBTIQ people of all ages and same sex couples and also people who were gender fluid and exploring, it was just glorious*”. Interview participant, international expert

In order to ensure that all new members of staff are LGBTQI+ positive, or at least are open to being trained in LGBTQI+ affirmation in care, it was suggested that care services advertise an explicit pro-LGBTQI+ ethos. As one interview participant stated:

*“Now on their website there is a proactive, ‘we don’t tolerate any kind of discrimination, we are fully inclusive we welcome LGBTQ people’, I mean its screaming, ‘don't work for us if you don’t like LGBTQ people’ because we do.”* Interview participant, international expert

Some participants suggested the need to diversify the workforce by hiring more LGBTQI+ care workers. Other participants, however, stated that the identity of the care provider was not so relevant as their dedication and level of training. Mandatory and comprehensive training was suggested by multiple participants, as people who were more biased towards LGBTQI+ people would most likely skip the training if it were not mandatory.

“ *I am concerned about an apparent absence of training specifically built into medical, nursing, and social care training in relation to sexuality and its impacts on older people because the attitudes towards older people are generally very poor in this country*.” Interview participant lesbian cis-gender woman.

When the topic of dedicated services was discussed, participants had differing views. Some did not like the idea of differentiated services and would prefer to be in a mixed setting. An international expert stated that lesbian women, particularly those who live separatist lives, often preferred women-only services rather than LGBTQI+-specific services. An interview participant stated that though he had never considered the idea of an LGBTQI+ specific service, that he was very interested in the idea.

*“It would feel very enticing to be with your own people, also that way you have people with whom you can talk. I do know that current seniors LGBTQ seniors in care facilities is that they find it very isolating, the heteronormativity of their peers. How do you have conversations...folks like us...today’s young people are so much more integrated… In our generation you lived such a life apart in many ways and it would be so nice to live with people who know what that is.”* Interview participant, gay trans-gender man.

***Understanding the variety of LGBTQI+ networks and life experiences.*** Heteronormative assumptions were reported to be problematic and embarrassing for many participants. Survey participants emphasized the need to understand and respect the variety of networks and life experiences of LGBTQI+ people. Many people noted that they are often assumed to be heterosexual or cisgender until told otherwise which can cause discomfort and puts the responsibility of disclosing gender and sexuality on the service user. As a survey participant stated:

“ *I'm okay with straight people caring for me and assume they would be tolerant - but the structures assume a heteronormative life*”. Survey participant, queer cis-gender woman. 59 years

One participant stated that she strongly disliked the fact that responsibility for " *coming out*” always rested on her. Instead, she stated that she would prefer it if people just asked her rather than assuming. As one survey participant stated:

“ *Older people are treated as though they have little or no interest in sexual relationships. In a heteronormative society, this means that they are generally assumed to be 'weakly heterosexual'. For individuals requiring personal care, this can be distressing*.” – Survey participant, lesbian trans-gender woman. 65 years

There is a need for non-nuclear family structures and friend networks to be respected and understood by professional dementia-care providers. It is important to avoid assumptions and ask questions about a person’s available network. As one interview participant stated:

“ *Those explicit questions about what social groups do we have now […] how can we maintain that without assuming that […] people will have a particular type of interest or hobby that people will engage in, and actually actively supporting peoples engagement with queer communities if they are engaged in those communities*. ” – Interview participant, international expert

It was suggested by an international expert, that an independent advocate should be triggered upon a dementia diagnosis, who could act in an older person’s best interests in cases where an individual’s social network was smaller, or in cases where unaccepting families of origin or other potentially exploitative people are acting against the best interest of the service user.

***Experiences specific to those who are transgender.*** Unlike with the experiences of sexual minorities, trans identity is often over-focused on in a healthcare context, which can both waste a service-user’s time when trying to focus on non-transgender related health issues and can feel uncomfortable and unnecessary.

“ *I sometimes don’t like how I am outed by default, by all sorts of specialists who don’t really need to know…why would they need to know, and I am uncomfortable so it’s more the opposite, I am not so happy about every care provider in whatever remote context knowing I’m trans, you know sometimes I think it’s not necessary*.” Interview participant, gay trans-gender man.

A transgender interview participant cited prior negative healthcare experiences as influencing his concern that he may be mistreated and humiliated in a care context,stating:

“ *My biggest concern I become very care dependent, would be to have my body mocked, or to be alienated, or that they would get sloppy with my medication regime. I'm honestly not even sure what medical recommendations would be about hormone treatment in high old age, because again our cohort are sort of a natural experiment…this kind of neglect of our particular situation*.” – Interview participant, gay trans-gender man.

With regards to transgender dementia care, an international expert noted that there are two opposing schools of thought about gender affirmation in dementia. One school of thought, which the participant was opposed to, was to rigidly affirm the gender as expressed by the transgender person, before their diagnosis with dementia. In many reported cases, transgender people with dementia can experience gender dysphoria and different gender identities can become more salient at different times, which can be confusing and distressing. She believed that to address someone as their previously preferred gender identity, whilst they are presenting or feeling like another would be “ *well-meaning coercion, but coercion none the less*”. Instead, this participant suggested the second school of thought, which is a more person-centred “ *take me as I am*” approach, in which care providers address a person as the gender that they are most comfortable with in that moment. 

### Consensus-based Needs and Recommendations

Following the analysis of the interview and survey data, the consensus meeting was held with ten key stakeholders (described in Phase Five above). There were ten core needs and sixteen recommendations derived from the data and literature gathered. The complete unranked list of needs and the complete unranked list of recommendations can be seen in the Extended data.

The final top ten need and recommendations, along with the associated rank score are presented in Table 7.

**Table 7.** Needs and Recommendations identified through the consensus process.

Rank	Top 10 Needs	Score	Rank	Top 10 Recommendations	Score
1	To feel respected and for your partner to feel respected.	9.6	1	At first contact with services/ at diagnosis, everyone should be given a multitude of resources including information about LGBTQIA+ services.	*7.25*
2	To feel safe in expressing your identity if you want to.	9.5	2	LGBTQIA+ older adults should have a choice between integrated and dedicated services.	6.63
3	To know that you, or your partner, are entering into a safe environment.	9.5	3	Integrated services with mandatory comprehensive training for staff should be available where dedicated services are unavailable.	6.63
4	To have dignity in all areas of treatment, especially end of life care.	9.4	4	LGBTQIA+ specific services for older adults and people with dementia should be introduced.	6.38
5	Care that values your needs as individuals and as LGBTQI or A+ people.	9.3	5	Services’ LGBTQIA+ inclusiveness and training should be auditable by a relevant health authority.	6.13
6	To be safe from abusive families of origin (if you have an abusive family of origin).	9.3	6	Service-users should be asked who they would like to help them in their care and decision making as their dementia symptoms progress	6.0
7	In a nursing home/ residential care setting, to be safe from homophobic/transphobic bullying/mistreatment from other residents.	9.1	7	Independent advocates for people with dementia should be triggered upon diagnosis. Advocates can work with people with dementia and their close networks to give them the care they desire most.	5.0
8	Not to feel pressured into expressing your identity if you don’t want to/ or don’t feel safe.	8.8	8	Training should include understanding differences in LGBTQIA+ networks and how to incorporate an individual’s network in care without making assumptions; as well as intervening with homophobic/ transphobic bullying/mistreatment from family of origin/other.	4.25
9	Provide specific trans* and intersex medical training for doctors and care staff working with older LGBTQIA+ people, to enable them to work safely with unfamiliar bodies.	8.8	9	When working with transgender people with dementia, care providers should address them as the gender they are presenting as in the current moment and not engage in any kind of coercion regarding their gender expression.	4.13
10	The need to support trans* people with dementia while also recognising the reality of biology and that some supports may require a focus on sex and not gender.	8.3	10	An explicitly LGBTQIA+ inclusive ethos message and visible displays of LGBTQIA+ acceptance should be clearly displayed in leaflets and webpages of dementia services. This must be accompanied by staff trained in LGBTQIA+ affirmative care.	3.0

## Discussion

This research has identified a prioritised, consensus-developed, and PPI-driven list of needs and recommendations for healthcare delivery for people with dementia from the LGBTQIA+ community. Having developed this list, the next crucial step is the implementation of these findings into practice to ensure we are delivering a human rights-based care for people with dementia, as recommended by the World Health Organization (2015).

The importance of maintaining identity came across in all phases of the research. For those living with dementia there is a duality in terms of managing dementia and also managing one’s own identity ( McParland & Camic, 2018). The conflict that people face in terms of who to disclose their identity to and in what context was evident in the findings, and echoes previous research describing the challenge of “ *giving yourself away vs. holding onto yourself*” ( McParland & Camic, 2018). With a diagnosis of dementia, it can also be difficult for people to remember who they told what to,that can be distressing.

Respect was another clear message that came from the research data. As well as respect for identity, respecting families of choice and including them in care decisions, when appropriate, was apparent from the research findings. Previous research has referred to relationships for people with dementia from the LGBTQIA+ community as “ *sheltered harbours*” where people feel safe and comfortable ( McParland & Camic, 2018). The focus on including family of choice in care decisions and plans came across clearly in this research. It can also be challenging for people to maintain healthcare regimes, such as long-term hormone therapy without assistance, and using the support systems that people already have in place has the potential to improve outcomes for people with dementia.

Safety when accessing services was also a priority for participants in this research. It is evident from previous research that avoidance of healthcare services can lead people to being admitted to residential care when it could have been avoided ( Westwood, 2016). The fear expressed by participants in becoming dependant on healthcare services because of possible neglect or mistreatment has been seen in earlier research ( Putney *et al.*, 2018). This deep-seated anxiety has been found to lead to identity concealment and chronic distress ( Putney *et al.*, 2018). Ensuring the people feel safe when accessing services should be fundamental. As older people from the LGBTQIA+ community do not feel safe (see the focus on safety in the “top 10” needs identified) this should be addressed immediately at a service-level.

There were conflicting views in relation to the need for dementia-specific services for LGBTQIA+ community. Even if not requested by all, participants agreed that it would be beneficial to have the choice to engage with these services as they are needed by some. The importance of welcoming, open, and non-judgemental services was identified as both a need and a recommendation. The importance of having an “ *explicit ethos*” was discussed at length and the need for visual representation of all types of older people in services, including sub-groups such as older LGBTQIA+ people from the Travelling community. Linked with this was mandatory training for healthcare professionals and the need to integrate this at the beginning of career training. The importance of this type of training being mandatory, integrated, and comprehensive was clear from the research data collected and has been reported elsewhere ( Fredriksen-Goldsen *et al.*, 2014; Nowaskie & Sewell, 2021).

### Limitations

The number of participants included in the research was small. This was anticipated by the research team and a number of steps were taken to ensure a consensus-based process- e.g the research contained multiple phases; the research was led by a representative PPI advisory group; the consensus meeting used purposive sampling to ensure representation across groups; and a number of recruitment avenues were used.

The questionnaire itself was lengthy and required a level of concentration that may have unintentionally excluded those with more severe dementia. Although the research included incomplete questionnaires (46.94% completion rate) and allowed for the questionnaire to be completed by or with a caregiver, there are likely people who were unable to take part because of this. The challenges posed by the COVID-19 pandemic limited the possibility of in-person data collection. Although the research team placed paper versions of the questionnaires in LGBTQIA+ community centres, many older people were sheltering at home and not attending these locations.

The questionnaire did not capture the views of caregivers (only 13.89% of the total sample) and it is suggested that further research looks at this cohort separately, as we know that this group often has fears about the future that are coloured by their own experiences of caregiving ( Price, 2011). We included caregivers in interview and consensus stages, but this is limited to a small number of caregivers.

Finally, the research team acknowledge that the term “older”, set for this research as 50+, will vary in terms ofethnicity and life expectancy due to health disparities. For instance, in 2016 only 3% of people from the Travelling community in Ireland were aged 65 or older and ageing in this community has been redefined as being aged 40+ as their life expectancy is 17% lower than the non-Travelling Irish community ( Gibney *et al.*, 2018). Future researchers may also consider allowing participants to decide if they identify as “older” rather than having a cut-off for the research.

## Conclusion

Although older LGBTQIA+ adults demonstrate strong resilience, many have significant worries about future, particularly in the context of dementia care. This research has provided a clear list of needs and recommendations that have been identified by the older LGBTQIA+ community as urgent and essential for improving healthcare access,safety and quality of life in care. It is vital that the staff in healthcare, voluntary, and community services working with older people are trained in understanding the needs of LGBTQIA+ older adults with dementia, and that services are explicitly welcoming and respectful when supporting LGBTQIA+ people with dementia and their care partners.

This research has identified key recommendations which may be used to further develop best practice in this area. Prior to this study, no research had been completed in Ireland to identify the needs and recommendations in this area in Ireland. Importantly, this research has had a strong PPI focus and has been directed by older LGBTQIA+ people. Throughout the work with PPI members and participants stressed the urgent need for the translation of this research into improved and more welcoming care for those from the LGBTQIA+ community.

## Data Availability

The data that support the findings of this study, including the questionnaire answers, and transcripts,are available on request from the corresponding author [S.M.H]. The data are not publicly available due to their containing information that could compromise the privacy of research participants. Due to the smaller sample of interview participants, and the specific and unique nature of some of the described life events of the participants that were paramount to analysis, de-identification is not sufficient to prevent possible recognition of the individuals. Open Science Framework: Dementia service needs and recommendations for LGBTQIA+ community. https://doi.org/10.17605/OSF.IO/P3UJE ( Hynes, 2022). This project contains the following extended data Paper version of National Survey (a copy of the questionnaire) Topic Guide (interviews with LGBTQI+ older adults) Document (Interview guide with experts) Consensus Event Annotated Agenda 9 ^th^ December 2021 (Topic guide – consensus meeting) Are you LGBTQIA+ and aged 50 or over (Sample social media and physical recruitment poster) SRQR_Checklist_dementia (SRQR checklist) Data are available under the terms of the Creative Commons Attribution 4.0 International license (CC-BY 4.0).

## Footnotes

^1^ The acronym LGBTQIA+ is used throughout this article to recognise the desires of people who identify as queer, questioning, intersex or asexual, who have experienced marginalisation within the LGBTQI+ community, to achieve increased visibility ( George, 2021). Please note that the acronym LGBTQI+ was used in the survey so resultantly this term is used in this section.
