# Supplementary material for: Developing consensus-based recommendations for the delivery of dementia services for the LGBTQIA+ community in the Republic of Ireland
Source: HRB Open Res. 2023 Feb 28;5:19. Originally published 2022 Mar 9. [Version 3] doi: 10.12688/hrbopenres.13505.3 (PMC11401990; doi:10.12688/hrbopenres.13505.3)
Supplement: Peer review history

# Reviewer report: Reviewer response for version 3

**DOI:** 10.21956/hrbopenres.14976.r34669

**Published:** 2024-09-14

**Authors:** Lars-Christer Hydén

**Affiliations:** 1 Linköping University, Linköping, Sweden

This manuscript is about the development of recommendations for the delivery of dementia services for the LGBTQIA+ community. The aim was to explore future care needs in the LGBTQIA+ community in relation to dementia care services, as well as to develop consensus-based recommendations. The study is based on a consensus process involving key stakeholders. A questionnaire was developed and tested, and then distributed nationally to persons who identified as members of the LGBTQIA+ community (or supported someone who were), were over 50 years of age and could provide informed consent. In total 49 responses were recorded. The questionnaires were analysed statistical and conceptually (open-ended answers). As a third step, six interviews were performed with international experts and two members of the LGBTQIA+ community. A thematic analysis of the interview was performed, and four main themes identified: anticipated concerns, the need for a LGBTQIA+ affirmative ethos and workforce as well as an understanding of the variety of the LGBTQIA+ community and its needs, and issues related to persons who are transgender. A review of international best practices was conducted and used together with the survey. From this analysis a preliminary list of needs and recommendations was created. This list was submitted to a group of key stakeholders. The list was revised and then re-submitted to the PPI group and finalized as a list of priorities.

 The manuscript is clearly written and makes an important contribution to the policy development concerning being members of the LGBTQIA+ community. I have some suggestions, mainly about clarifications of the methods used and recruitment of participants.

 (1) 49 responses to the national questionnaire is fairly low. Maybe the reasons for the low number could be discussed. Table 1 should also contain the absolute numbers not just percentage (as all other tables show).

 (2) Some more information about the respondents and their relation to the total community is needed. A central concern is if the respondents can be seen as representative of the whole community - or if some group or groups are left out.

 (3) It would be good with some more information about the interviews. Why just six interviews, and why these interviewees? Why not interviews with many more members from the LGBTQIA+ community? How were the interviewees recruited?

 (4) It is unclear what the basis is for the results presented in the section “Analysis of qualitative data- interviews and survey”. Is it based only on the interviews, or is based on the interviews and the survey? If the survey data was included this needs to be described and clarified how it was used.

 (5) How were the participants in the PPI advisory group recruited? What do “purposeful sampling strategy” mean in this context? Could the PPI group be seen as representative for the LGBTQIA+ community as a whole?

Is the work clearly and accurately presented and does it cite the current literature?

Yes

If applicable, is the statistical analysis and its interpretation appropriate?

Yes

Are all the source data underlying the results available to ensure full reproducibility?

Yes

Is the study design appropriate and is the work technically sound?

Yes

Are the conclusions drawn adequately supported by the results?

Yes

Are sufficient details of methods and analysis provided to allow replication by others?

Partly

Reviewer Expertise:

Dementia studies

I confirm that I have read this submission and believe that I have an appropriate level of expertise to confirm that it is of an acceptable scientific standard.

# Reviewer report: Reviewer response for version 3

**DOI:** 10.21956/hrbopenres.14976.r34667

**Published:** 2023-07-10

**Authors:** Neil Drummond

**Affiliations:** 1 University of Calgary, Calgary, Alberta, Canada

This paper presents the findings from a mixed method study of health and social service needs of aging LGBTQIA2S people living with dementia. Methods applied include an internet and mailed quantitative survey, semi-structured interviews, a consensus conference using a nominal group approach and the identification of needs and recommendations for fulfilling them. The study was strongly guided by principles of participatory community-based research.

 I enjoyed reading this report. The study was well done and thorough and I think the findings will be useful in improving care for people with dementia in the Irish context, if policy makers are convinced by it. I think there must be concern that the sample sizes involved are not really large enough to strongly support the evidence being presented, but the authors acknowledge this and I don't think this alone is reason for rejecting the report for indexing and dissemination.

 I do have some niggling issues that I think should be addressed, as follows.

 1.         In Table 1 14% of respondents were LGTBQ2 etc. living with dementia. This translates to 6-7 people. Despite the fact that doing so highlights the small samples I think the numbers involved should be reported alongside the percentages. This is science after all and we should be honest.

 2.         Tables 2 and 3 are said to indicate that older LGTBQ2 people become more isolated from their community, but that's true for most people as they age. Some acknowledgement of this would help readers judge the impact of the tendency.

 3.         Despite the above, Fig 1 appears to show that most respondents had maintained community engagement, at least to their own satisfaction.

 4.         Fig 3 contains duplication of the "Relevant in some situations" response.

 5.         Table 5 displays responses to a "Lifetime experience of abuse" item. This is probably misleading unless contextualized by presenting general population norm data. Everybody suffers abuses of some kinds over a lifetime.

 6.         As above, for Table 6. And the item "I was denied or provided inferior care" could be read as "I was denied inferior care or provided inferior care", and is hence ambiguous.

 7.         In the qualitative data section the international expert's comment about the crucifix sounds equivalent to judging a book by its cover. It may not signify what she assumes it does.

 8.         In the "Limitations" section the authors state that the questionnaire was returned by 13.89% of care partners. As noted above, Table 1 reports that 13.89% (or 14%) of respondents were LGTBQ2 living with dementia, and 2.78% (2 people?) were care partners. I would round up these percentages.

 9.         In the questionnaire, the responses to the Q15 "use of services" items leap from 1/y to "never", which seems a bit unreasonable and may have led to serious undercounting. An intervening <1/y before the "never" might have been useful. The same goes for Q16.

 10.       In the Topic Guide, the "Many people in the survey commented that .... Has this happened to you?" structure is clearly leading.

Is the work clearly and accurately presented and does it cite the current literature?

Yes

If applicable, is the statistical analysis and its interpretation appropriate?

Not applicable

Are all the source data underlying the results available to ensure full reproducibility?

Yes

Is the study design appropriate and is the work technically sound?

Yes

Are the conclusions drawn adequately supported by the results?

Yes

Are sufficient details of methods and analysis provided to allow replication by others?

Yes

Reviewer Expertise:

Dementia epidemiology, primary care, population health surveillance using primary care EMR data.

I confirm that I have read this submission and believe that I have an appropriate level of expertise to confirm that it is of an acceptable scientific standard, however I have significant reservations, as outlined above.

# Reviewer report: Reviewer response for version 2

**DOI:** 10.21956/hrbopenres.14924.r33136

**Published:** 2022-12-22

**Authors:** Miriam Galvin

**Affiliations:** 1 Academic Unit of Neurology, School of Medicine, Trinity College Dublin, Dublin, Ireland

Could respondents answer more than one response in the community participation question?

 Tables 2 and 3 LGBTQI **A **in the label but not in the response categories, or text.

*Only two activities were attended by participants in the majority of cases, these were LGBTQI+ groups and Cultural/arts groups. - *Not sure what this means

 Percentages in Figure 2 and 3 would be useful.

 Figure 3 is LGBT identity interchanged with LGBTQ!A or + etc?

*There were ten core needs and sixteen recommendations derived from the data and literature gathered - *How? 

 Some typos throughout.

 The authors' revisions are good. There are no major, significant changes required but feel still some work needed.

Is the work clearly and accurately presented and does it cite the current literature?

Partly

If applicable, is the statistical analysis and its interpretation appropriate?

Partly

Are all the source data underlying the results available to ensure full reproducibility?

Yes

Is the study design appropriate and is the work technically sound?

Partly

Are the conclusions drawn adequately supported by the results?

Partly

Are sufficient details of methods and analysis provided to allow replication by others?

Partly

Reviewer Expertise:

NA

I confirm that I have read this submission and believe that I have an appropriate level of expertise to confirm that it is of an acceptable scientific standard, however I have significant reservations, as outlined above.

# Response

**Published:** 2023-02-08

**Authors:** Sinéad Hynes

**Affiliations:** NUI Galway, Ireland

Thank you for re-reviewing this manuscript. We appreciate the time and effort involved. We have addressed all of the areas raised in the newest version of the manuscript. In summary it is as follows:

- Could respondents answer more than one response in the community participation question?
  - It was only possible to provide one answer here. This note has been added for clarification. 
- Tables 2 and 3 LGBTQIA in the label but not in the response categories, or text.
  - This has been changed, thank you. 
- " *Only two activities were attended by participants in the majority of cases, these were LGBTQI+ groups and Cultural/arts groups*". - Not sure what this means.
  - This meant that participants reported taking part in no more than two activities. We have deleted this sentence as it is not clear and not important additional information to have in the text. 
- Percentages in Figure 2 and 3 would be useful – percentages provided for both. 
  - Percentages are now available for both. 
- Figure 3 is LGBT identity interchanged with LGBTQ!A or + etc?
  - This has been changed. 
- There were ten core needs and sixteen recommendations derived from the data and literature gathered - How? 
  - A paragraph has now been added that we hope clarifies this.
- Some typos throughout.
  - We hope we have now spotted all of the typos and made required corrections. Thank you. 

# Reviewer report: Reviewer response for version 1

**DOI:** 10.21956/hrbopenres.14732.r32684

**Published:** 2022-09-09

**Authors:** Lyn Phillipson, Keryn Johnson

**Affiliations:** 1 Health and Society, Arts, Social Sciences and Humanities Faculty, University of Wollongong, Wollongong, NSW, Australia; 2 School of Health and Society, Arts, Social Sciences and Humanities Faculty, University of Wollongong, Wollongong, Australia

The aims of the paper were to 1) Identify the future needs of older LGBTQIA+ people and their care partners living in Ireland have in relation to dementia care service delivery (note that the abstract has LGTBQIA+) and 2) develop consensus-based recommendations for dementia service provision in Ireland. It should be noted that the research itself is orientated to the people with dementia and does not give recommendations on the needs of caregivers.

 The research is topical and needed however there are some fairly major issues that need to be resolved in how the research is described in this paper. There is a lot of data being reported on in the papers, yet not enough detail on methods to allow the reader to really assess the integrity of the findings. Further detail on methods is required. There is little justification of the methods except perhaps for the modified Delphi. Further justification is required.

 The authors may need to consider the potential benefits of breaking this research up into perhaps 2 or even 3 papers: survey, qualitative, and overall consensus process to address the level of detail that is required to ensure the rigour of the article is not in question

**Overall recommendation: Major Revisions required**

****

 Page 3

 The background literature is current and relevant.

 However, a few more references supporting the lived experience of LGBTQIA+ would be appropriate.

 Please revise and tighten the narrative and improve punctuation over the next few pages (the use of short and longer dashes e.g. Phases 1 & 2 headings).

 There are also grammatical mistakes throughout the paper that require corrections (‘s’ where there should be, missing ‘the’s, incorrect were/was, the use of ‘&’ within the text) that need to be addressed.

 Please provide further details of the make up of the PPI.

 Please provide further details about what the survey was about – what was its content and how did it change throughout the Delphi process.

 The statement ‘thorough review of the literature’ is not sufficient evidence of support for the design of the survey. Please provide more details on the process used and what results it provided.

 It says PPI decided on inclusion of items etc – did the researchers collaborate on this, or was this totally PPI decision-making?

 You state the survey was piloted in Phase 1 – but don’t mention it in Phase 1. Please clarify.

 Page 4

 Not sure what you mean by interviews through an online platform. Zoom? Email?

 How was Phase 4 used within the research? Please provide further detail on the process or what you asked the experts. Were these results incorporated into the interview results? This is not clear.

 Please provide more specifics with regards to participant backgrounds - ‘Voluntary and healthcare backgrounds’ is not sufficient.

 Please provide further details with regards to how the results of Phases 2,3,4 were presented to the group. Also on how you present the needs and recommendations at the same group – who developed these? You?

 Who were presented the results for member checking? Please specify.

 Please give details of the PPI group -somewhere in the paper.

 How many in the pilot group?

 Page 5

 Survey data collection ceased – not sampling ceased. Please correct.

 Please describe the data analysis more clearly i.e. quantitative data was analysed i.e. descriptive statistics, open-text (qualitative data) was analysed via content analysis (although you don’t really present the findings as per a content analysis – more like a thematic so I am not sure what process was used. Please clarify).

 Quite a detailed qualitative analysis and then only 2 themes? Please justify/explain further.

 Explain what you mean by verification of data integrity. How?

 What were the multiple observer/observations that you did?

 How did the PPI work with the qualitative data?

 Please consider reporting demographics in a table. This would improve clarity and ease of reference for readers.

 In academic writing it is convention to spell out a number if it starts a sentence. Please correct.

 Page 6

 Please work on introducing your survey results better - please provide further background into what you asked and to improve the readers capacity to understand the results.

 Figure 1 is not really a good graphic to present these results. Please revise.

 Also is it not clear if people could only tick one box, as it adds up to 100%? A bar chart might be better.

 Page 7

 These tables are easier to understand but I don’t understand why means and standard deviations are here – they are not appropriate to this data. Please revise.

 Page 8

 Again these pie charts are overly big and use a lot of space, and difficult to read in black and white– consolidate these into a table.

 Figure 3 label – missing QAI+.

 Page 9

 The open text results need more introduction. Please provide.

 ‘Like in prior research’ – please provide references to support this statement.

 Please attribute the quotes in the paper with the appropriate participant identifiers to ensure adequate context. This is usual practice and should be followed e.g. Carer, age 55.

 Please make further comment on the results in the tables – e.g. what were the highest forms of discrimination.

 Page 10

 Lack of protection ‘from’ discrimination. Please correct.

 If reporting all these percentages, a table is better.

 Again the pie chart uses up too much room for the information it imparts – try to consolidate with other data.

 Page 11

 You said 8 interviews earlier – now 6? Please correct and ensure accuracy.

 I think there could be some sub-themes here. Please clarify and improve depth of responses.

 Page 12

 ‘Additional note’ is not a theme, and should be labelled differently.

 Page 13

 I think your headings are getting a bit lost in the text. Please revise.

 Please give specific numbers for people in your consensus process e.g. former carers (n=3).

 Still not quite sure how the recommendations came from the data and who did it.

 Page 14

 Needs and recommendations should be put into a table or box or something to set them apart – they are the crux of your paper.

 Page 15

 Discussion is ok. But further information is required to address what this research adds to current knowledge specifically.

 Page 6

 Normally you do not reference within a conclusion, as you are just summarising what you have already said. Please correct.

Is the work clearly and accurately presented and does it cite the current literature?

Partly

If applicable, is the statistical analysis and its interpretation appropriate?

No

Are all the source data underlying the results available to ensure full reproducibility?

No source data required

Is the study design appropriate and is the work technically sound?

Partly

Are the conclusions drawn adequately supported by the results?

Partly

Are sufficient details of methods and analysis provided to allow replication by others?

No

Reviewer Expertise:

Dementia, qualitative research methods

We confirm that we have read this submission and believe that we have an appropriate level of expertise to confirm that it is of an acceptable scientific standard, however we have significant reservations, as outlined above.

# Response

**Published:** 2022-10-24

**Authors:** Sinéad Hynes

**Affiliations:** NUI Galway, Ireland

Dear Dr Phillipson and Dr Johnson,

 Many thanks for your review and comments. We have considered each comment and have amended the manuscript considering your review. We have also answered some of your queries and comments here:

**Comment 1: The aims of the paper were to 1) Identify the future needs of older LGBTQIA+ people and their care partners living in Ireland have in relation to dementia care service delivery (note that the abstract has LGTBQIA+) and 2) develop consensus-based recommendations for dementia service provision in Ireland. It should be noted that the research itself is orientated to the people with dementia and it does not give recommendations on the needs of caregivers.**

**The research is topical and needed, however, there are some fairly major issues that need to be resolved in how the research is described in this paper. There is a lot of data being reported on in the papers, yet not enough detail on methods to allow the reader to really assess the integrity of the findings. Further detail on the methods is required. There is little justification of the methods except perhaps for the modified Delphi. Further justification is required. The authors may need to consider the potential benefits of breaking this research up into perhaps 2 or even 3 papers: survey, qualitative and overall consensus process to address this level of detail that is required to ensure the rigour of the article is not in question.**

 Response: The authors considered detailing the results of the survey, interviews, and consensus process individually, however, we believe that this would remove vital context from each individual paper. We concluded that our findings were more valuable if presented as a whole, particularly because the results and findings from the survey and the interviews, for example, were very closely tied and intrinsically linked. The aim of the research was to identify the needs and recommendations and we feel it necessary to report on the entire process together in order to do this. However, we have made some formatting changes and substantial restructuring of the manuscript, particularly the results section, in relation to your review and comments from other reviewers which should help to simplify the presentation of our results.

 We have noted the typo in the abstract relating to the acronym LGBTQIA+ and changed it as such. Please note that we use both LGBTQI+ and LGBTQIA+ in the manuscript- as addressed in Reviewer 1 response. We have also added further justification to our methods section which will hopefully clarify the rigor of this article. We have done this while also trying to reduce the length of the manuscript, as requested by reviewers.

 We also acknowledge that further research should be oriented towards creating recommendations and core needs for the caregivers of LGBTQIA+ people with dementia in our limitations section as this would be beneficial to further establishing international best practices for this group of service users.

**Comment 2: The background literature is current and relevant. However, a few more references supporting the lived experiences of LGBTQIA+ people would be appropriate.**

 In the interest of reducing the length of the manuscript we did not included additional reference to the lived experience of LGBTQIA+ people. We feel that the main areas are covered in the background and adding additional discussion would make the background overly-lengthy. We hope this is acceptable to you.

**Comment 3: Please revise and tighten the narrative and improve punctioation over the next few pages (the use of short and longer dashes e.g. Phases 1&2 headings) There are also grammatical mistakes throughout the paper that require corrections (s’ where there should be, missing ‘the’s’ and the incorrect were/was, b and use of ‘&’ within the text.) this needs to be addressed.**

 Response: We hope that all grammatical and punctuation errors have been addressed now.

**Comment 4: Please provide further details of the make-up of the PPI.**

 The PPI members were not participants and as a result, it is not appropriate to provide detailed description of the group. They were, however, all older people from the LGBTQIA+ community or carers for an older person from the LGBTQIA+ community.

**Comment 5: Please provide further details about what the survey was about – what was its content and how did it change throughout the Delphi process?**

 Response: The survey provided information on participants, previous experiences with healthcare use, general discrimination, community participation, how important they viewed their sexual or gender identity to be in a healthcare context, and how participants viewed their identity. The survey also utilized text-response questions in which participants could elaborate on their experiences surrounding accessing care and what they believed their future use of healthcare might look like.

 We are not sure if the reviewer is referring to changes to the survey during the piloting phase rather than the Delphi process? Several small edits and clarifications were made at this stage, but nothing that changed the overall content.

 Data from the survey were analysed descriptively and via content analysis and were used to inform the initial needs and recommendations for presentation to the consensus meeting. Further information on how these recommendations and needs were developed have been added to the manuscript. The questionnaire itself can be viewed in the linked Extended data (at the end of the manuscript before the References section).

**Comment 6: The statement ‘thorough review of the literature’ is not sufficient evidence of support for the design of the survey. Please provide more details on the process used and what results it provided.**

 We did not create the survey. The questionnaire that we used has been developed by researchers in the US and used on an American group of participants. This survey was adapted for use in Ireland. We have added to our description of the survey design. The reference for the original survey is included in the paper.

**Comment 7: It says PPI decided on the inclusion of items – did the researchers collaborate on this or was this totally PPI decision-making?**

 Response: This was a collaboration between the researchers and the PPI group. Items from the questionnaire (Fredriksen-Goldsen & Kim, 2017) were adapted and the addition of dementia-specific questions or phrases used (such as the inclusion of the word queer) was also discussed with the PPI group.

**Comment 8: You state the survey was piloted in Phase 1 – but don’t mention it in Phase 1. Please clarify.**

 Response: This has been clarified in Phase 1. This section has been restructured and we hope it is now easier to follow.

**Comment 9: Not sure what you mean by interviews through an online platform. Zoom? Email?**

 Response: Zoom was used for all interviews.

**Comment 10: How was Phase 4 used within the research? Please provide further detail on the process or what you asked the experts. Were these results incorporated into the interview results? This is not clear.**

 Interviews with international experts were interviewed to provide information on best practice. They were included in the thematic analysis. We have expanded somewhat on this in the manuscript.

**Comment 11: Please provide more specifics with regards to participant backgrounds – Voluntary and healthcare backgrounds is not sufficient.**

 Response: We have added more to the backgrounds in relation to whom we were recruiting, however, we cannot include any potential identifiers in this report.

**Comment 12**: **Please provide further details with regards to how the results of phases 2,3,4 were presented to the group. Also on how you present the needs and recommendations at the same group – who developed these? You?**

 Response: Phases 2,3,and 4 were presented to the consensus group via a zoom presentation. A PowerPoint presentation was used and each step of the research was described along with the results from each of these phases a discussion was then opened up to the group following the presentation. This description can be found in both our results section under the heading Consensus process and in our method section under the heading ‘Phase 5’. We used Slido to facilitate the voting. Participants could see the results “in real-time” during the meeting using this online tool.

 A detailed description of how the initial needs and recommendations were developed has also been included in light of this comment.

**Comment 13: Who were presented the results for member checking? Please clarify.**

 Response: The consensus group were presented with the results for checking. We have clarified this in the manuscript.

**Comment 14: Please give details of the PPI group – somewhere in the paper.**

 Please see Comment 4 for this.

**Comment 15: How many in the pilot group?**

 Seven people completed the pilot (this data was not included in the results).

**Comment 16: Survey data collection ceased – not sampling ceased. Please correct**

 Response: This was a grammatical error on our part and has been changed.

**Comment 17: Please describe the data analysis more clearly i.e. quantitative data was analysed i.e. descriptive statistics, open text (qualitative data) was analysed via content analysis (although you don’t really present the findings as per content analysis – more like thematic so I am not sure what process was used. Please Clarify).**

 The data analysis has now been included across each of the Phases for ease of reading. We hope this has adequate information. We can provide additional information if required for any of the phases of analysis. We have not provided an lengthy description because of the length of the manuscript.

**Comment 18: Quite a detailed qualitative analysis and then only 2 themes? Please justify/explain further.**

 We have presented this slightly differently now as four themes (and additional theme was added on request of reviewer 1).

**Comment 19: Explain what you mean by verification of data integrity.**

 The data was screened when exported into Excel. The data was cleaned and the source data was checked to ensure there were no omissions or errors.

**Comment 20: What were the multiple observer/observations?**

 This term has been removed as it is somewhat confusing. We had meant that we are multiple data collection methods- e.g. survey and interviews.

**Comment 21: How did the PPI work with the qualitative data?**

 The PPI group advised on the creation of the topic guides.

**Comment 22: Please consider reporting demographics in a table. This would improve clarity and ease of reference for readers.**

 This has now been done and can be seen in Table 1.

**Comment 23: Please work on introducing your survey results better and open text results need more introduction.**

 More information has now been provided and the manuscript has been re-structured to ease understanding.

**Comment 24: Please revise Figure 1. Is it not clear if people could only tick one box, as it adds up to 100%? A bar chart might be better.**

 We have changed the other figures (from pie-charts) that were presented in the manuscript. We are, however, reluctant to change Figure 1 as PPI advisors have expressed a desire to keep this figure as is for ease of understanding. People could only tick one box here- this is why it adds up to 100%.

**Comment 25: Please attribute quotes in the paper**

 This has now been included for each quote.  

**Comment 26: Please make further comment on the results in the tables.**

 We have included some further discussion. This is not exhaustive and the tables are included for further reference.

**Comment 27: I think there could be some sub-themes here**

 We have broken this down further into four separate themes that we believe improves the reporting of the results, while staying true to the analysis process.

**Comment 28: Needs and recommendations should be put into a table or box or something to set them apart – they are the crux of your paper.**

 These are now included as a table.

**Comment 29: Discussion is ok. But further information is required to address what this research adds.**

 This has now been added to the end of the manuscript. We have also deleted the reference, as requested.

 We would like to thank you again for your contributions, which we believe have greatly increased the standard and rigor of this article.

 Kind regards,

 Megan H. Oglesby & Sinéad M. Hynes

# Reviewer report: Reviewer response for version 1

**DOI:** 10.21956/hrbopenres.14732.r32612

**Published:** 2022-09-02

**Authors:** Miriam Galvin

**Affiliations:** 1 Academic Unit of Neurology, School of Medicine, Trinity College Dublin, Dublin, Ireland

The aim of this research was to identify the future needs that older LGTBQIA+ people and their care partners living in Ireland have in relation to dementia care service delivery; and to develop consensus-based recommendations for dementia service provision in Ireland. This is a relevant and interesting area for exploration. And translating their work into recommendations is a welcome addition.

 This study employed multiple methods – survey, interviews and consensus meeting.

 Background includes relevant literature on LGBT+ people from Ireland and on an international scale.

 Ethical approval for this work was granted, and clearly stated. Reporting standards were used, with PPI involvement as per good practice.

 General:

- A lot of data and information are presented in this paper. The authors could present the survey findings or the interview findings in individual papers and the consensus and recommendations in another paper for example.
- While it is useful to detail the whole process that was undertaken, and report the findings from each phase in this paper, if the whole work is included could the authors condense the information presented?

Specific issues:

- Could the authors explain the addition of QIA+ to the more common LGBT+ for readers to understand?
- Could the authors say a bit more about why dementia services in particular are the focus of their work? Are the requirements for those services different to healthcare services more generally?
- Are the 6 phases described (pp 3-4) part of the consensus process or the research process?
- It may be clearer for readers if the ‘data collection tools’ were linked with each of the phases identified rather than in the following subsection (p4)?
- Page 4 *Sampling stopped after no more responses were recorded in the survey for a period of two weeks*. – does that mean the survey was closed to data being collected, how is this sampling?
- More information on the survey is needed:
- How was the survey undertaken, on-line? Postal?
- How are the authors defining completion rate? How many people were asked to do the survey? How many responded? How were paper versions of surveys returned to authors? How were the survey data uploaded/downloaded etc?
- Could the authors describe how and why they used content analysis from the survey data? It is not clear to this reader how these data were analysed – is it the quotes presented?
- Could the authors clarify why they present means and standard deviations in the tables e.g., Table 1, what does a mean of 1.27 indicate?

How is the reader to differentiate the quotes in the survey section from those in the interview analysis section?

 Interview data (p11) as a heading – should this be analysis of interview data?

 Could the authors comment on why they did 6 interviews? Were more than 6 recruited etc?

 Why were international experts included in the interviews?

 Page 12 – additional note on transgender experiences and identity – the heading type is similar to those used for the themes – suggest this should be changed or else it is a 3 ^rd^ theme?

 Page 13 Consensus process How were 10 key stakeholders identified? Were there criteria used to include people?

 How were the core needs and recommendations derived from the data (before ranking)?

 Page 16 Limitations: *This indicates that there may be older people who have not disclosed to anyone at this point that have been excluded from the data*. Not clear what this means, how do the authors know this?

 Did the authors consider adapting the survey to make it shorter?

 Looking at the National Survey document– was there an ‘A’ as in LGBTQIA+?

Is the work clearly and accurately presented and does it cite the current literature?

Partly

If applicable, is the statistical analysis and its interpretation appropriate?

Partly

Are all the source data underlying the results available to ensure full reproducibility?

Yes

Is the study design appropriate and is the work technically sound?

Partly

Are the conclusions drawn adequately supported by the results?

Partly

Are sufficient details of methods and analysis provided to allow replication by others?

Partly

Reviewer Expertise:

NA

I confirm that I have read this submission and believe that I have an appropriate level of expertise to confirm that it is of an acceptable scientific standard, however I have significant reservations, as outlined above.

# Response

**Published:** 2022-10-24

**Authors:** Sinéad Hynes

**Affiliations:** NUI Galway, Ireland

Dear Dr Galvin

 Many thanks for your review and comments. We have considered each comment and have amended the manuscript in light of your review. We have also answered some of your queries and comments here:

**Comment 1: A lot of data and information are presented in this paper. The authors could present the survey findings or the interview findings in individual papers and the consensus and recommendations in another paper for example. While it is useful to detail the whole process that was undertaken and report the findings from each phase in this paper, if the whole work is included could the authors condense the information presented?**

 Response: The authors considered detailing the results of the survey, interviews and consensus process individually, however we believe that this would remove vital context from each individual paper. We concluded that our findings were more valuable if presented as a whole particularly because the results and findings from the survey and the interviews, for example, were very closely tied and intrinsically linked.

 We have, however, made formatting changes and substantial restructuring and revision of the manuscript, particularly the results section, in relation to your review and comments from other reviewers which should help to simplify the presentation of our results. We have also reduced the text and the length of the quotes in some places which has reduced the text by approximately 1500 words.

**Comment 2: Could the authors explain the addition of QIA+ to the more common LGBT+ for readers to understand?**

 Response: The addition of QIA+ was discussed with our PPI group, as the authors have noted the increase in the popularity of this acronym due to its increased inclusiveness in comparison to LGBT (George, 2021). Although we considered using LGBT+, with the plus symbol signaling further inclusiveness, our PPI group members preferred the specific inclusiveness to Queer/Questioning, Intersex and Asexual people who are becoming increasingly visible within the community and whose needs have been marginalised within the LGBT community (George, 2021). We also believed that it would specifically highlight our aim to include Intersex, asexual and people who identify as queer or questioning into our study.  We have inserted a small explanatory section in the manuscript as a footnote to justify this decision.

**Comment 3: Could the authors say a bit more about why dementia services in particular are the focus of their work? Are the requirements for those services different to healthcare services more generally?**

 Response: Dementia services were the focus of this study because prior studies have found that some members of the LGBTQIA+ community are more likely to develop dementia due to secondary reasons such as being more likely to experience social isolation, or being more likely to develop HIV or depression which increases their risk factor for developing dementia. Research in the area of LGBTQIA+ dementia has increased in the last number of years, however there were no specific data relating to LGBTQIA+ people with dementia and their access to care, except for the open letter (Roe et al., 2020) in which more research into the accessibility of care for older LGBTQIA+ older adults was called for. Additionally, as in Ireland there were no existing LGBTQIA+-specific dementia service at the time of writing the manuscript, we believed that it would be beneficial to specifically examine care provision for this demographic. Many of these findings may be generalisable to older LGBTQIA+ adults accessing care services in Ireland whether they have dementia or not.

**Comment 4: Are the 6 phases described (pp 3-4) part of the consensus process or the research process?**

 Response: The six phases described are a part of the consensus process, but this is also the overall research process that was followed by the research team. The consensus process is the method used (with a consensus meeting forming a phase of this).

**Comment 5: It may be clearer for readers if the ‘data collection tools’ were linked with each of the phases identified rather than in the following subsection (p4)**

 Response: This has been amended and much of this section has been merged with the phases section.  

**Comment 6: Sampling stopped after no more responses were recorded in the survey for a period of two weeks. – Does that mean the survey was closed to data being collected, how is this sampling?**

 Response: This was a grammatical error on our part and has been amended.

**Comments 7-12: More information on the survey is needed:**

 The survey was postal and online based. However no postal responses were received. Questionpro was the online survey platform, on which we calculated how many people completed the entire survey as opposed to partial completion, this is what we are referring to when we write about the “completion rate”. Unfortunately, we cannot know how many people were asked to complete the survey as we used a number of social media platforms as well as dementia and LGBTQIA+ organisations for recruitment purposes. Forty-nine people responded to the survey.

 Content analysis was used on the survey responses because the survey included long answer questions. However, the responses to these questions tended to be quite short, varying in length between a single paragraph to a single sentence. The authors believed it would be helpful to analyse the frequency of coded answers throughout the survey, as opposed to recurring themes within a single answer. An explanation of why we used content analysis on these data has been added to the manuscript.

 Quotes in the survey sections were quotes from the long-answer survey questions and quotes presented under the heading of ‘Interview Data’ were from the Interviews. However, this has been restructured as the long-answer questions were used to create themes from the content analysis. The themes derived from this analysis were very closely related to the themes that were developed during the thematic analysis of the interview data and therefore they are now be presented together. To help the reader differentiate between quotes we have included a descriptor of what type of participant created the quote (i.e. – interview participant or – survey participant).

 The inclusion of standard deviations and means was an error and they have been removed.

**Comment 13: Interview data (p11) as a heading – should this be analysis of interview data?**

 This has been amended.

**Comment 14: Could the authors comment on why they did six interviews? Were more than six recruited etc?**

 Response: There were a total of eight interviews. Six interviews were with international experts and two were with LGBTQIA+ older adults. Older LGBTQIA+ adults were recruited for an interview through social media and relevant organizations and therefore we cannot comment on how many people in this cohort were approached for recruitment. The six international experts were recruited via email, more than six were approached but six responded and followed through with the interview. We have clarified this in the manuscript.

**Comment 15: Why were international experts included in the interviews?**

 Response: International experts were included to gather more information surrounding the development of international best practice. International experts were not invited to take part in the consensus meeting- including ranking of items (as described in Phase 5).

**Comment 16: Additional note on transgender experiences and identity – the heading type is similar to those used for the themes – suggest this could be changed or else it is a third theme?**

 Response: Thank you- the authors have substantially restructured this section and this has now been included as an overall theme.   

**Comment 17: Consensus process: How were the 10 key stakeholders identified? Were the criteria used to include people?**

 Ten key stakeholders, who consisted of LGBTQIA+ people with dementia, LGBTQIA+ older adults, former caregivers of LGBTQIA+ older adults, and people who have worked with LGBTQIA+ older adults and/or people with dementia took part in the consensus meeting. A purposeful sampling strategy was used to ensure a diverse group with varied experiences and backgrounds. We included all members of the PPI group (as this was already a representative group) and the additional members were invited from dementia organisations in Ireland. Criteria were the same as for the survey (described in Phase 2).  

**Comment 18: How were the core needs and recommendations derived from the data before ranking?**

 Response: Core needs and recommendations were predominantly derived from the themes and subthemes that were developed from the interviews and long-answer survey responses, as well as from the literature on the topic. For instance, where a problem clearly exists in the themes a recommendation to solve that problem was either developed or taken from the participants’ recommendations. In some cases, such as the recommendation to provide LGBTQIA+-relevant information leaflets along with other more generalised dementia leaflets, the recommendation was derived directly from an interview source as this recommendation, in particular, was already being conducted in some dementia organisations in the UK but not in Ireland, and we believed this was relevant in relation to the development of international best practice.

**Comment 19: This indicates that there may be older people who have not disclosed to anyone at this point that have been excluded from the data. Not clear what this means, how do the authors know this? ** 

 Response: This has been removed from the manuscript as it may cause confusion. We had meant that all of our participants had previously disclosed their sexuality or gender identity to another person at least once. This indicates that our survey did not capture the experiences of any older LGBTQIA+ person who has not “ *come out of the closet*”. We have tried not to use this phrase in the manuscript but include it here to help clarify what we had meant.

**Comment 20: Did the authors consider adapting the survey to make it shorter?**

 Response: Yes, we had. We consulted extensively with the PPI group on this and a decision was made to include all items that are currently there but to accept incomplete questionnaires (those with missing data). The survey did use skip logic, which would have shortened the length of time to undertake the survey to an extent.

**Comment 21: Looking at the National Survey document – was there an ‘A’ as in LGBTQIA+ ?**

 Yes, this is correct. Although asexual people are often included in the “+” that was in the survey, this was not explicitly stated. We have changed it accordingly in the results section to avoid confusion. We have not changed throughout the entire manuscript as some of the research presented- including the recommendations and needs explicitly included those who identify as asexual.

 We would like to thank you again for your contributions, which we believe have greatly increased the standard and rigor of this article.

 Kind regards,

 Megan H. Oglesby & Sinéad M. Hynes
